# Type I interferon autoantibodies are associated with systemic immune alterations in patients with COVID-19

**DOI:** 10.1126/scitranslmed.abh2624

**Published:** 2021-09-22

**Authors:** Monique G. P. van der Wijst, Sara E. Vazquez, George C. Hartoularos, Paul Bastard, Tianna Grant, Raymund Bueno, David S. Lee, John R. Greenland, Yang Sun, Richard Perez, Anton Ogorodnikov, Alyssa Ward, Sabrina A. Mann, Kara L. Lynch, Cassandra Yun, Diane V. Havlir, Gabriel Chamie, Carina Marquez, Bryan Greenhouse, Michail S. Lionakis, Philip J. Norris, Larry J. Dumont, Kathleen Kelly, Peng Zhang, Qian Zhang, Adrian Gervais, Tom Le Voyer, Alexander Whatley, Yichen Si, Ashley Byrne, Alexis J. Combes, Arjun Arkal Rao, Yun S. Song, Gabriela K. Fragiadakis, Kirsten Kangelaris, Carolyn S. Calfee, David J. Erle, Carolyn Hendrickson, Matthew F. Krummel, Prescott G. Woodruff, Charles R. Langelier, Jean-Laurent Casanova, Joseph L. Derisi, Mark S. Anderson, Chun Jimmie Ye, on behalf of the UCSF COMET consortium

**Affiliations:** 1 Department of Genetics, University of Groningen, University Medical Center Groningen, 9713AV Groningen, Netherlands.; 2 Institute of Human Genetics, University of California, San Francisco, San Francisco, CA 94143, USA.; 3 Division of Rheumatology, Department of Medicine, University of California, San Francisco, San Francisco, CA 94143, USA.; 4 Medical Scientist Training Program, University of California, San Francisco, San Francisco, CA 94143, USA.; 5 Tetrad Graduate Program, University of California, San Francisco, San Francisco, CA 94143, USA.; 6 Diabetes Center, University of California, San Francisco, San Francisco, CA 94143, USA.; 7 Department of Biochemistry and Biophysics, University of California, San Francisco, San Francisco, CA 94158, USA.; 8 Laboratory of Human Genetics of Infectious Diseases, Necker Branch, INSERM U1163, Necker Hospital for Sick Children, 75015 Paris, France.; 9 University of Paris, Imagine Institute, 75015 Paris, France.; 10 St. Giles Laboratory of Human Genetics of Infectious Diseases, Rockefeller Branch, Rockefeller University, New York, NY 10065, USA.; 11 ImmunoX Initiative, University of California, San Francisco, San Francisco, CA 94143, USA.; 12 Department of Medicine, University of California, San Francisco, San Francisco Medical Service, San Francisco Veterans Affairs Health Care System, San Francisco, CA 94121, USA.; 13 School of Medicine, University of California, San Francisco, San Francisco, CA 94143, USA.; 14 Chan Zuckerberg Biohub, San Francisco, CA 94158, USA.; 15 Zuckerberg San Francisco General, San Francisco, CA 94110, USA.; 16 Department of Laboratory Medicine, University of California, San Francisco, San Francisco, CA 94143, USA.; 17 Division of HIV, Infectious Disease and Global Medicine, Department of Medicine, University of California, San Francisco, San Francisco, CA 94143, USA.; 18 Laboratory of Clinical Immunology and Microbiology, Division of Intramural Research, National Institute of Allergy and Infectious Diseases (NIAID), National Institutes of Health (NIH), Bethesda, MD 20814, USA.; 19 Vitalant Research Institute, San Francisco, CA 94118, USA.; 20 Vitalant Research Institute, Denver, CO 80230, USA.; 21 University of Colorado School of Medicine, Aurora, CO 80045, USA.; 22 Geisel School of Medicine at Dartmouth, Lebanon, NH 03755, USA.; 23 Department of Electrical Engineering and Computer Sciences, University of California, Berkeley, Berkeley, CA 94720, USA.; 24 Department of Biostatistics, University of Michigan, Ann Arbor, MI 48109, USA.; 25 Department of Pathology, University of California, San Francisco, San Francisco, CA 94143, USA.; 26 UCSF CoLabs, University of California, San Francisco, San Francisco, CA 94143, USA.; 27 Department of Statistics, University of California, Berkeley, Berkeley, CA 94720, USA.; 28 Division of Hospital Medicine, University of California, San Francisco, San Francisco, CA 94143, USA.; 29 Division of Pulmonary, Critical Care, Allergy and Sleep, Department of Medicine and the Cardiovascular Research Institute, University of California, San Francisco, San Francisco, CA 94143, USA.; 30 Division of Infectious Disease, Department of Medicine, University of California, San Francisco, San Francisco, CA 94143, USA.; 31 Howard Hughes Medical Institute, New York, NY 10065, USA.; 32 Division of Endocrinology and Metabolism, Department of Medicine, University of California, San Francisco, San Francisco, CA 94143, USA.; 33 Departments of Epidemiology and Biostatistics and Bioengineering and Therapeutic Sciences, University of California, San Francisco, San Francisco, CA 94143, USA.; 34 Bakar Computational Health Sciences Institute, University of California, San Francisco, San Francisco, CA 94143, USA.; 35 Parker Institute for Cancer Immunotherapy, San Francisco, CA 94129, USA.

## Abstract

Neutralizing autoantibodies against type I interferons (IFNs) have been found in some patients with critical coronavirus disease 2019 (COVID-19), the disease caused by severe acute respiratory syndrome coronavirus 2 (SARS-CoV-2). However, the prevalence of these antibodies, their longitudinal dynamics across the disease severity scale, and their functional effects on circulating leukocytes remain unknown. Here, in 284 patients with COVID-19, we found type I IFN–specific autoantibodies in peripheral blood samples from 19% of patients with critical disease and 6% of patients with severe disease. We found no type I IFN autoantibodies in individuals with moderate disease. Longitudinal profiling of over 600,000 peripheral blood mononuclear cells using multiplexed single-cell epitope and transcriptome sequencing from 54 patients with COVID-19 and 26 non–COVID-19 controls revealed a lack of type I IFN–stimulated gene (ISG-I) responses in myeloid cells from patients with critical disease. This was especially evident in dendritic cell populations isolated from patients with critical disease producing type I IFN–specific autoantibodies. Moreover, we found elevated expression of the inhibitory receptor leukocyte-associated immunoglobulin-like receptor 1 (LAIR1) on the surface of monocytes isolated from patients with critical disease early in the disease course. LAIR1 expression is inversely correlated with ISG-I expression response in patients with COVID-19 but is not expressed in healthy controls. The deficient ISG-I response observed in patients with critical COVID-19 with and without type I IFN–specific autoantibodies supports a unifying model for disease pathogenesis involving ISG-I suppression through convergent mechanisms.

## Interfering with interferons

A subset of patients diagnosed with coronavirus disease 2019 (COVID-19) present with autoantibodies specific to type I interferons (IFNs). However, the systemic impacts of type I IFN–specific autoantibodies are not fully understood. Here, van der Wijst *et al.* longitudinally evaluated the relationship between type I IFN–specific autoantibody abundance and changes to the immune system of individuals with COVID-19. Using single-cell transcriptomics, the authors found that the presence of type I IFN autoantibodies correlated with reduced type I IFN–stimulated gene (ISG) expression in patients with critical COVID-19. Reduced ISG expression, in turn, correlated with increased expression of the inhibitory receptor, leukocyte-associated immunoglobulin-like receptor 1 (LAIR1), on monocytes. Together, these findings suggest that early evidence of type I IFN autoantibodies and increased LAIR1 expression may help distinguish severe cases of COVID-19.

## Teaser

Type I interferon autoantibodies in patients with COVID-19 are associated with poor interferon responses and increased LAIR1 abundance in leukocytes.

## INTRODUCTION

The coronavirus disease 2019 (COVID-19) pandemic has led to the infection of at least 192 million individuals worldwide and more than 4.1 million deaths. A perplexing aspect of severe acute respiratory syndrome coronavirus 2 (SARS-CoV-2) pathogenesis is the extreme clinical heterogeneity of infected individuals, with about 15% of symptomatic patients and less than 10% of infected individuals presenting with severe forms of the disease, as defined by dyspnea, pulmonary infiltrates on lung imaging, and low blood oxygen saturation (*1*–*4*). Overall, 26.8% of patients who are hospitalized develop critical disease defined as category 7 on the National Institutes of Health (NIH) ordinal scale, requiring mechanical ventilation (*5*). These patients are at the greatest risk for poor outcome and place the largest burden on the health care system. Despite increasing vaccine availability, some vulnerable individuals may develop critical disease before and even perhaps despite vaccination, especially in the context of emerging highly transmissible, more virulent, and antigenically distinct variants of SARS-CoV-2 isolates (*6*–*11*). Thus, there is a need to disentangle the immunological consequences of SARS-CoV-2 infection and the underlying immunological causes of critical COVID-19 to stratify patients early in their disease course and to target treatments accordingly.

Evidence is emerging that genetic and immunological features that predate SARS-CoV-2 infection could play an unexpected pathogenic role in severe disease (*12*). Among patients with critical COVID-19, these features include inborn errors of type I interferon (IFN)–mediated immunity (*13*, *14*) and the production of autoantibodies against type I IFNs (*15*, *16*). These autoantibodies, which seldom occur in healthy controls (with frequencies less than 0.3%) and have not been found in asymptomatically infected individuals, are observed in at least 10% of patients with critical COVID-19 (*15*, *17*–*19*). The causal effects of autoantibodies against type I IFNs on COVID-19 severity has been supported by their documentation before infection and their frequent occurrence in patients with genetic disorders, such as autoimmune polyglandular syndrome type 1 (APS-1) (*20*–*22*).

However, it remains to be determined whether autoantibodies to type I IFNs occur in patients with COVID-19 who do not require mechanical ventilation, whether they fluctuate longitudinally during the disease course, and what their consequences are on the composition and phenotypes of circulating leukocyte subsets. Furthermore, few studies have examined circulating leukocytes over the course of SARS-CoV-2 infection (*23*, *24*) or have compared with patients presenting with similar respiratory manifestations requiring hospitalization due to other causes (*25*). Insights into how the natural innate and adaptive immunity longitudinally evolve in response to SARS-CoV-2 infection, in both anti–type I IFN autoantibody-positive and autoantibody-negative cases, may enable the early identification of patients who are likely to develop life-threatening COVID-19 and the discovery of mechanisms that can be targeted by therapy.

## RESULTS

### Anti–IFN-α2 antibodies are reproducibly detected in patients with COVID-19 but seldomly detected in the general population

**Fig. 1.**
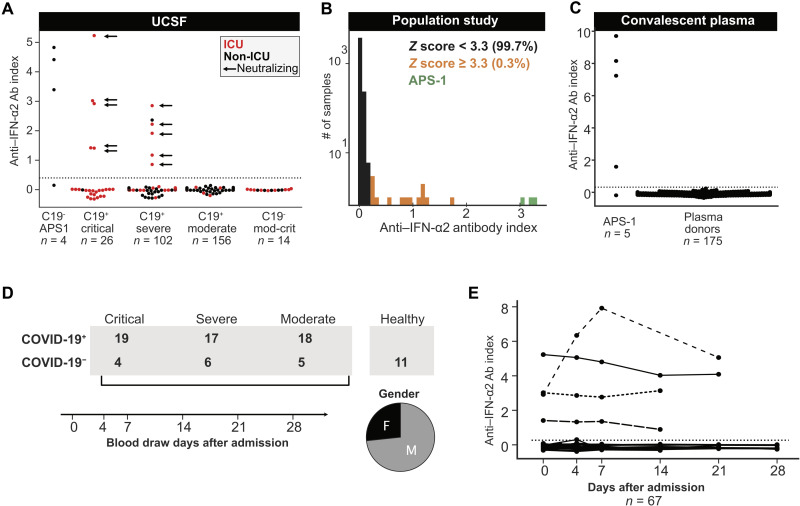
Detection of anti–IFN-α2 antibodies is positively associated with COVID-19 disease severity. (**A**) Anti–IFN-α2 antibody (Ab) index values (*y* axis) for 4 APS-1 patients, 26 critical COVID-19^+^ (C19^+^) cases, 102 severe COVID-19^+^ cases, 156 moderate COVID-19^+^ cases, and 14 moderate-severe COVID-19^−^ (C19^−^) cases, separated by disease severity and colored by hospitalization status. Positive samples were tested for neutralization against IFN-α2 and IFN-ω (see fig. S1), with arrows indicating those samples with partial or full neutralization ability. The dotted line indicates 6 SDs above healthy control mean. (**B**) Distribution of the anti–IFN-α2 index values across 4041 individuals in a community cohort from the San Francisco Mission District. (**C**) Anti–IFN-α2 index values (*y* axis) for five additional APS-1 patients and 175 CCP donors from the Vitalant Blood Center. (**D**) Timeline of blood draws for participants in the COVID-19 Multi-Phenotyping for Effective Therapies (COMET) cohort. Disease status, severity, and gender breakdowns are also shown. (**E**) Anti–IFN-α2 Ab index values over days since first hospitalization for 53 hospitalized COVID-19^+^ and 14 COVID-19^−^ COMET samples. For 2 of 69 COMET samples, anti–IFN-α2 titers were not assessed.

Given the recent description of neutralizing type I IFN autoantibodies in greater than 10% of critical COVID-19 cases, we sought to determine the frequency of these antibodies in San Francisco in a total of more than 4,500 individuals divided over the following: (i) SARS-CoV-2–positive individuals that span the NIH COVID-19 severity scale (*26*), (ii) a largely asymptomatic community population, and (iii) convalescent serum samples from patients previously infected with SARS-CoV-2. We first determined the frequency of autoantibodies to the type I IFN, IFN-α2, in 284 patients with confirmed SARS-CoV-2 infection using a radioligand binding assay (RLBA). These patients were categorized using the NIH ordinal scale, with those scoring between 1 and 4 classified as moderate, those scoring 5 or 6 as severe, and those scoring 7 as critical (Table 1). Of the 284 patients, 53 were enrolled in the COVID-19 Multi-Phenotyping for Effective Therapies (COMET) cohort (Table 2 and data file S1) and 231 were enrolled from Zuckerberg San Francisco General Hospital and Trauma Center (ZSFG) (Table 3 and data file S2). We also tested 4 samples from individuals negative for COVID-19 with an APS-1 diagnosis as positive controls and 14 samples from individuals negative for COVID-19 without an APS-1 diagnosis as negative controls (Fig. 1A). The 284 patients ranged in age from 0 to 90+ years, were 69% male, had at least one positive SARS-CoV-2 polymerase chain reaction (PCR) test, and varied in disease severity (Tables 2 and 3 and data files S1 and S2). We found the prevalence of anti–IFN-α2 autoantibodies to be 5 of 26 (19%) in critical, 6 of 102 (6%) in severe, and 0 of 156 in moderate disease (Fig. 1A; see fig. S1 for the neutralizing capacity of these antibodies in the anti–IFN-α2–positive patients; *P* < 0.01, Fisher’s exact test comparing severe-to-critical to moderate cases). The positive patients were aged 28 to 72 years (mean = 55.7, std = 12.2), and 9 of 11 (82%) were male. The prevalence of anti–IFN-α2 in critical COVID-19 and the tendency of positive patients to be of the male sex and of advanced age are consistent with previously published descriptions (*15*).

**Table 1.** Categorization of moderate, severe, and critical patients in the COMET and ZSFG cohorts. ECMO, extracorporeal membrane oxygenation; n/a, not applicable.

**NIH ordinal scale**	**WHO criteria**	**Categorization: COMET cohort**	**Categorization: ZSFG cohort**
1	Not hospitalized, no limitations on activities	n/a	Moderate
2	Not hospitalized, limitation on activities or requiring home oxygen	n/a	Moderate
3	Hospitalized, not requiring supplemental oxygen—no longer requires ongoing medical care	Moderate	Moderate
4	Hospitalized, not requiring supplemental oxygen—requiring ongoing medical care	Moderate	Moderate
5	Hospitalized, requiring supplemental oxygen	Severe	Severe (oxygenation information not available)
6	Hospitalized, on noninvasive ventilation or high-flow oxygen devices	Severe	
7	Hospitalized, on mechanical ventilation or ECMO	Critical	Critical

**Table 2.** Demographics of the COMET cohort. Demographics and clinical characteristics of patients from the COMET cohort, broken down by anti–IFN-α2^+^ COVID-19^+^, anti–IFN-α2^−^ COVID-19^+^, and COVID-19^−^ patients. RLBA, radioligand binding assay.

**Characteristic**	COVID-19^+^ anti–IFN-α2^−^ (*n* = 50)*	**COVID-19^+^ anti–IFN-α2^+^ (RLBA** **value > 6 SDs above healthy** **controls) (*n* = 4)**	**COVID-19^−^ (*n* = 15)†**
Mean age (SD)	58.1 (16.8)	56.3 (11.8)	61.5 (14.2)
Sex			
Male	37	4	7
Female	13	0	8
Race			
White or European	13	0	7
Asian	9	1	3
Native Hawaiian/Other Pacific Islander	3	0	0
Black/African-American	1	0	1
Other/Multiple races	22	3	2
Unknown	2	0	2
Ethnicity			
Hispanic/Latino	20	3	1
Non-Hispanic/Latino	30	1	11
Unknown	0	0	3
Duration hospitalization (SD)	18.3 (15.6)	22.3 (8.4)	7.5 (5.1)
Died			
Yes	6	1	4
No	44	3	11

*One of 50 not tested for anti–IFN-α2.

†One of 15 not tested for anti–IFN-α2.

**Table 3.** Demographics of the ZSFG cohort. Demographics and clinical characteristics of patients from the ZSFG cohort, including comparison across anti–IFN-α2–positive and anti–IFN-α2–negative patients. Significance values were determined using Fisher’s exact test (FE), except in the case of continuous distributions (length of stay), where a Kolmogorov-Smirnov (KS) test was used.

**Characteristic**	**COVID-19^+^** **anti–IFN-α2^−^** **(*n* = 224)**	**COVID-19^+^ anti–** **IFN-α2^+^ (RLBA** **value > 6 SDs** **above healthy** **controls) (*n* = 7)**	***P* value**
Mean age (SD)	50.3 (15.9)	55.3 (13.3)	
Sex			
Male	144	6	0.46 (FE)
Female	78	1	
Unknown	2	0	
Race			
Black or African- American (8%)	19	0	1 (FE)
White (6%)	12	0	1 (FE)
Asian (6%)	12	1	0.35 (FE)
Other, including Hispanic or Latino ethnicity (78%)	178	5	1 (FE)
Not reported (2%)	3	1	0.13 (FE)
Ethnicity			
Hispanic or Latino (75%)	170	5	0.68 (FE)
Non-Hispanic or Latino (25%)	54	2	
Hospital admission	103	7	0.15 (FE)
Noninvasive oxygenation or more	79	6	0.12 (FE)
ICU	48	6	0.02 (FE)
Mechanical ventilation	31	4	0.04 (FE)
Pressors	25	5	0.006 (FE)
Death	7	2	0.04 (FE)
Mean length of stay (SD)	5.6 (10.7)	28 (30.0)	0.0005 (KS)
Men	5.5 (9.6)	30.2 (32.3)	0.003 (KS)
Women	5.4 (12.3)	15 (*n* = 1)	0.3 (KS)

We next examined a community cohort collected during a study of SARS-CoV-2 transmission in San Francisco (Fig. 1B, Table 4, and data file S3) (*27*). The cohort consisted of 4,041 individuals aged 4 to 90 years of European (36%), Hispanic/LatinX (33%), Asian/Pacific Islander (9%), Black/African American (2%), and other or unknown (20%) ancestry. In this cohort, a total of 13 anti–IFN-α2–positive individuals (0.32%) were identified. Of these, five were male, six were female, and two were of unknown gender. Positive samples were identified across all represented ethnic groups. None of the participants who were confirmed positive for past or present SARS-CoV-2 infection (117 of 3,851 by serology and 64 of 3,758 by PCR) were positive for anti–IFN-α2 antibodies, and all were ambulatory or asymptomatic at the time of testing. These data are consistent with the previously reported absence of autoantibodies in patients with ambulatory COVID-19 (*15*). Our results also confirm the low frequency of anti–IFN-α2 antibodies in individuals independent of, and likely before, infection with SARS-CoV-2. In addition to assessing the presence of anti–IFN-α2 antibodies in San Francisco community cohorts, we analyzed aliquots of convalescent plasma (CCP) from a central blood bank supplier, encompassing 175 unique plasma donors who had recovered from SARS-CoV-2. Compared with five additional individuals diagnosed with APS-1, we found that none of the donors tested positive for anti–IFN-α2 autoantibodies (Fig. 1C). This latter cohort suggests that these potentially harmful autoantibodies are rare or absent in the supply from convalescent donors.

**Table 4.** Demographics of the community cohort. Demographics of patients from the community cohort, broken down by anti–IFN-α2–positive and anti–IFN-α2–negative individuals. Significance values were determined using Fisher’s exact test.

**Characteristics**	**Anti–IFN-α2^−^ (*n* = 4028)**	**Anti–IFN-α2^+^ (*z* score > 3.3,** ***P* = 0.005) (*n* = 13)**	***P* value**
Mean age (SD)	41.0 (15.9)	51.5 (20.3)	
Sex			
Male	1811	5	0.77
Female	1731	6	
Unknown	486	2	
Race/ethnicity			
Hispanic or Latino (33.6%)	1354	4	1
White or European (36.2%)	1458	2	0.15
Asian or Pacific Islander (8.6%)	347	1	1
Two or more races (2.8%)	113	2	0.05
Black or African-American (2.0%)	79	1	0.23
Other (3.6%)	143	1	0.38
Unknown (13.2%)	534	2	0.69
Antibody test result			
Positive	117	0	
Not detected	3723	11	
Unknown	188	2	
PCR test result			
Positive	64	0	
Not detected	3684	10	
Unknown	280	3	
Antibody + PCR testing			
Positive for one or both tests	154	0	
Negative or unknown for both	3874	13	

### Single-cell epitope and RNA sequencing of peripheral blood mononuclear cells enables assessment of systemic immune associations to COVID-19 and the presence of type I IFN autoantibodies

We next sought to identify the associations of COVID-19 status and the presence of anti–IFN-α2 autoantibodies with the composition, transcript abundance, and surface protein abundance of circulating leukocytes. For this, we leveraged the COMET cohort where peripheral blood mononuclear cells (PBMCs) and serum were longitudinally collected from 69 hospitalized patients presenting with COVID-19 symptoms (67 of 69 patients overlapped with those for whom anti–IFN-α2 autoantibodies were assessed; Fig. 1A), of whom 54 were positive (COVID-19^+^) and 15 were negative (COVID-19^−^) for SARS-CoV-2, in addition to 11 healthy controls (Fig. 1D). Of the COVID-19^+^ cases, 18 presented with moderate disease, 17 with severe disease, and 19 with critical disease according to the NIH severity scale (*26*) at the time of hospitalization (Tables 1 and 3 and data files S1 and S4). For 8 of 54 COVID-19^+^ patients, the severity changed over the course of hospitalization, of whom 6 improved and 2 worsened. The studied hospitalized patients were ethnically diverse, skewed older than the general population (mean = 59, range = 25 to 90), and were predominantly male (47 men and 22 women) (Fig. 1D). Although all COVID-19^−^ cases presenting with symptoms concerning for COVID-19 tested negative for SARS-CoV-2, many were infected with common respiratory pathogens confirmed by metagenomic sequencing (data file S1). Among the 67 of 69 COMET patients assessed for anti–IFN-α2 autoantibodies, 4 of 19 (21%) of the critical COVID-19 cases, none of the moderate to severe cases, and none of the COVID-19^−^ cases were positive for anti–IFN-α2 antibodies (Fig. 1A). All four cases had anti–IFN-α2 antibodies at the earliest time of sampling, and the concentration of anti–IFN-α2 antibodies remained stable for three of four cases across their disease course (Fig. 1E).

To profile the circulating immune response, we collected about 200 PBMC samples from up to four longitudinal time points: 0, 4, 7, and 14 days since hospitalization. Multiplexed single-cell epitope and transcriptome sequencing (muxCITE-seq) was performed across nine pools of genetically distinct samples to simultaneously measure mRNA abundances transcriptome-wide and surface protein abundances of 189 markers from the same cell (fig. S2 and data file S5). A total of 971,550 cell-containing droplets were sequenced, and 600,929 cells remained in the final dataset after quality control and removal of doublets, platelets, and red blood cells. Genetic demultiplexing using Freemuxlet resulted in an average of 3020 cells per sample (fig. S3A).

### Critical COVID-19 is characterized by increased frequencies of plasmablasts and classical monocytes

**Fig. 2.**
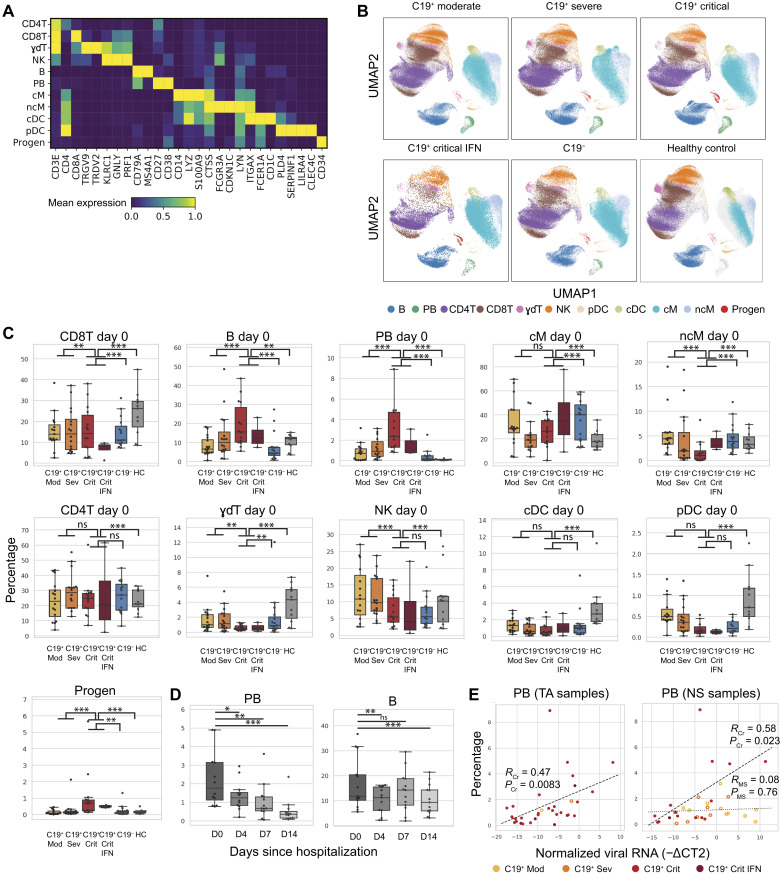
Shifts in circulating leukocyte composition are observed in samples isolated from individuals with critical COVID-19. (**A**) Marker genes are shown for each of the 11 cell types identified including CD4^+^ T cells (CD4T), CD8^+^ T cells (CD8T), γδ T cells (γδT), natural killer cells (NK), B cells (B), plasmablasts (PBs), classical monocytes (cM), nonclassical monocytes (ncM), conventional DCs (cDC), plasmacytoid DCs (pDC), and CD34^+^ hematopoietic progenitors (Progen). (**B**) UMAP projections of PBMCs are shown for donors separated by COVID-19 status and severity. Cells are colored by type. (**C**) Box plots of cell type percentages (*y* axis) by COVID-19 status and severity on day of hospital admission (D0). Each dot represents the percentage of a specific cell type per donor. Statistical comparisons between cells from all COVID-19^+^ critical donors (including the anti–IFN-α2 autoantibody donors) and healthy controls, COVID-19^−^ donors, or combined COVID-19^+^ moderate-severe donors are shown. (**D**) Box plots show the percentages of PB and B (*y* axis) in patients with COVID-19 over days 0, 4, 7, and 14 since hospitalization (D0, D4, D7, and D14). Data in (C) and (D) are presented as median and 25th and 75th percentiles. Statistically significant differences are presented using Holm’s multiple-testing corrected, permutation-based *P* values: *P < 0.05, **P < 0.01, and ***P < 0.001; ns, not significant. (**E**) Scatter plot of normalized SARS-CoV-2 viral RNA abundances (*x* axis) versus percentage of PB (*y* axis) was quantified in donor-matched single-cell PBMC data. RNA was measured by RT-qPCR in tracheal aspirates (left) or nasal swabs (right) as inverse ΔCT (inverse Ct difference between viral E gene and human RNase P gene). *R*, Pearson correlation in critical (*R*_Cr_) or moderate-severe (*R*_MS_) cases.

We compared the frequencies of 11 cell types between COVID-19^+^ cases, COVID-19^−^ cases, and healthy controls, as well as within COVID-19^+^ cases separated by severity. The assessed cell types were defined by their transcriptomic profiles and included plasmablasts (PBs), B cells (B), CD4^+^, CD8^+^, and γδ T cells (CD4T, CD8T, and γδT), natural killer cells (NKs), conventional and plasmacytoid dendritic cells (cDC and pDC), classical and nonclassical monocytes (cM and ncM), and hematopoietic progenitor cells (Progens) (Fig. 2A). We first confirmed that muxCITE-seq–derived estimates of lymphocyte and monocyte frequencies were correlated with complete blood count measurements reported in the electronic health record from the same donor within 2 days (Pearson *R*_monocyte_ = 0.59, *P* = 7.2 × 10^−105^; Pearson *R*_lymphocyte_ = 0.57, *P* = 8.2 × 10^−55^; fig. S3B). Qualitatively, COVID-19^+^ cases exhibited shifts in the uniform manifold approximation and projection (UMAP) space of circulating leukocytes, particularly of myeloid cells, that were not confounded by processing batch and pool (Fig. 2B and fig. S3C). Comparing critical COVID-19^+^ cases to healthy controls, we observed statistically significant changes in frequencies for every cell type, including prominent increases in the frequencies of cM, B, and PB (cM: median change +10.0%, differential proportion analysis permutation *P* < 10^−5^; B: +2.7%, *P* = 2.1 × 10^−3^; PB: +2.1%, *P* < 10^−5^) and decreases in the frequencies of CD8T, γδT, NK, cDC, and pDC (CD8T: −15.4%, *P* < 10^−5^; γδT: −3.9%, *P* < 10^−5^; NK: −4.7%, *P* < 10^−5^; cDC: −2.2%, *P* < 10^−5^; pDC: −0.6%, *P* = 3.0 × 10^−4^). The frequencies of CD8T, γδT, pDC, and PB in moderate and severe cases were between those observed in critical cases and healthy controls (Fig. 2C, fig. S3D, and data file S6). The frequency of CD8Ts was even lower, and the frequency of cMs was even higher in critical COVID-19^+^ cases with detectable anti–IFN-α2 antibodies than those without (CD8T: −5.8%, *P* < 10^−5^; cMs: +8.3%, *P* = 0.034) (Fig. 2C and data file S6). Changes in frequencies of B, PB, and ncMs were also significantly different between critical COVID-19^+^ cases and COVID-19^−^ hospitalized patients (B: +10.4%, *P* < 10^−5^; PB: +2.1%, *P* < 10^−5^; ncM: −2.4%, *P* < 10^−5^), suggesting that these effects are not likely explained by hospitalization in general (Fig. 2C and data file S6). For the 14 COVID-19^+^ donors for whom all four time points were available, we observed decreases in the frequencies of B and PB cells over time (median change D0 versus D14; B: −3.7%, *P* = 6.0 × 10^−5^; PB −0.8%, *P* < 10^−5^) and increases in the frequencies of cM and ncMs (D0 versus D14: cM +5.7%, *P* < 10^−5^; ncM +2.5%, *P* < 10^−5^) for both days since hospitalization and days since onset of first symptoms (Fig. 2D; fig. S3, E and F; and data file S6). These longitudinal changes normalized toward frequencies observed in healthy controls. The exception was the frequency of cMs, which appeared to further increase from those observed in healthy controls. Previously, the frequency of PBs had been observed to correlate with COVID-19 disease severity (*28*, *29*) and to diminish upon recovery (*24*). We observed a positive correlation between viral RNA abundances from tracheal aspirate samples, collected from mostly critical cases, and PB frequency (in critical cases: Pearson *R* = 0.47, *P*_adjusted_ = 0.0083) (Fig. 2E); both the PB frequencies (Fig. 2D) and viral RNA abundances decreased over time (fig. S4A). As expected from the high correlation between viral RNA abundances measured in tracheal aspirates and nasal swabs from the same donors (fig. S4B; Pearson *R* = 0.94), we replicated the high correlation between viral RNA and PB frequency in nasal swab samples of critical cases (Pearson *R* = 0.58, *P*_adjusted_ = 0.023) (Fig. 2E). However, in moderate and severe cases, no association was found (Pearson *R* = 0.08, *P*_adjusted_ = 0.76) (Fig. 2E). On the contrary, for none of the other cell types, a correlation between cell type frequency and viral titers was identified, independent of disease severity (fig. S4, C and D). Together, these results suggest coordinated dynamic changes of host humoral immunity and viral load over the course of hospitalization in critical cases but do not implicate a specific cause. Nevertheless, a recent study by Stephenson *et al.* (*29*) confirmed that viral RNA abundance is an independent contributing factor to cell type proportion changes observed in patients with COVID-19. Overall, these analyses revealed shifts in cell type composition specific to COVID-19, between patients of varying disease severity, and over time. The general comparable composition of circulating leukocytes among patients with COVID-19^+^ of varying severity with and without anti-IFN autoantibodies (fig. S3D) suggests the presence of a broader, conserved mechanism underlying critical disease, such as additional IFN-related pathology, particularly in the autoantibody-negative patients.

### Critical COVID-19 is marked by deficient type I IFN–stimulated gene expression in myeloid cells early in disease

**Fig. 3.**
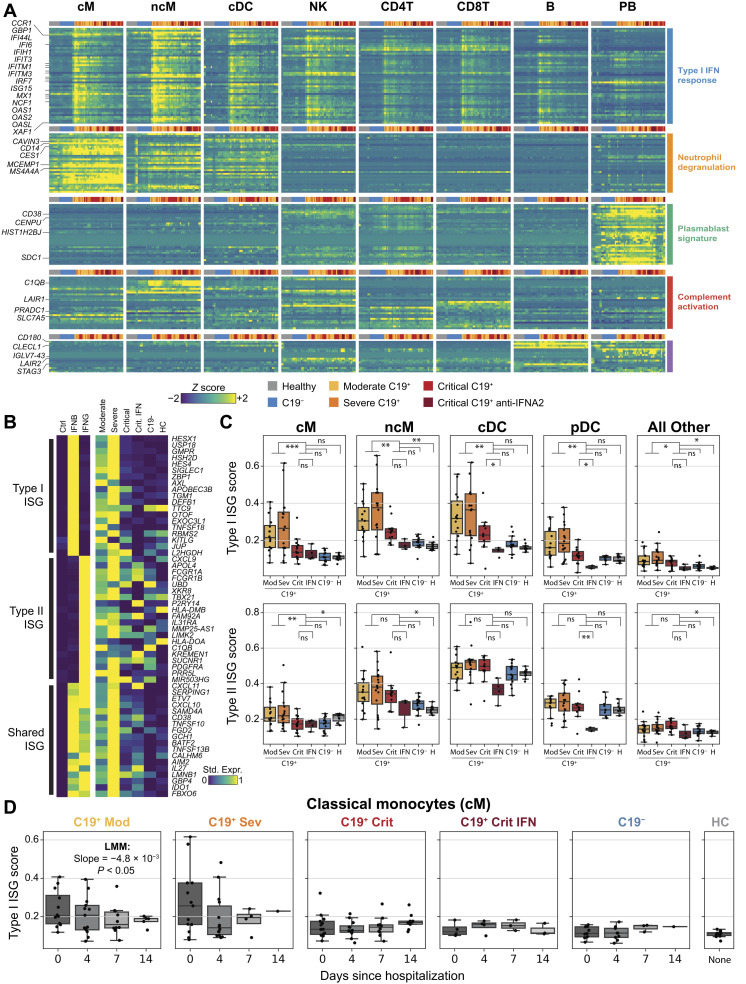
The degree of activation of the ISG-I response differs among the COVID-19 severity scale. (**A**) The heatmap shows 161 differentially expressed genes at day 0 [FDR < 0.01, |log(fold change)| > 1] in at least 1 of 11 cell types. CD4^+^ T cells (CD4T), CD8^+^ T cells (CD8T), NK cells, B cells (B), PB, cM, ncM, and cDC are shown. Each row represents a gene, and each column is the average expression of the genes in a particular sample across all cells of a specific type. Samples are grouped by both case control status and COVID-19^+^ severity. Expression is row standardized. Genes are grouped by cluster, with the enriched clusters annotated. (**B**) Matrix plots showing the expression of shared, type I–specific, and type II–specific ISGs in cM. The shared and specific ISGs were defined using an orthogonal scRNA-seq dataset containing PBMCs stimulated with IFN-β and IFN-γ or left unstimulated (Ctrl) (left). The expression of these same gene set was then plotted in the COMET cohort separated by case control status and disease severity (right). (**C**) ISG-I and ISG-II scores (*y* axis) at day 0 across four myeloid cell types and pseudobulk of all other cell types, separated by case control status and disease severity (*x* axis). Box plots show median and 25th and 75th percentile. (**D**) ISG-I scores (*y* axis) over the course of disease for healthy controls and COVID-19^−^ and COVID-19^+^ cases in cMs. COVID-19^+^ cases are separated by severity and the presence of anti–IFN-α2 antibodies. Inset shows significant results of linear mixed model (LMM) testing for changes over time. *P < 0.05, **P < 0.01, and ***P < 0.001.

To further characterize cell type intrinsic differences associated with COVID-19 and the presence of anti–IFN-α2 antibodies, we compared mRNA and surface protein abundances between COVID-19^+^ cases, COVID-19^−^ cases, and healthy controls for each cell type. We identified 161 genes [false discovery rate (FDR) < 0.05, log_2_FC > 1] whose transcripts were differentially up-regulated between COVID-19^+^ cases at day 0 and healthy controls in at least one cell type (Fig. 3A, fig. S5A, and data file S7). *K*-means clustering of the 161 differentially expressed genes aggregated for each of the 11 cell types at day 0 identified five clusters, including a cluster (cluster 1) of genes enriched for type I IFN signaling and viral response primarily expressed in myeloid cells [gene set enrichment analysis (GSEA): type I IFN signaling pathway, permutation *P* < 10^−5^], a cluster (cluster 2) enriched for neutrophil degranulation (GSEA: neutrophil degranulation, *P* < 10^−5^), a cluster (cluster 3) of immunoglobulins (Igs) and PB activation markers, and a cluster (cluster 4) enriched for complement activation in ncMs (GSEA: complement activation, *P* = 0.026) (data file S8). Given the heterogeneous expression of the IFN signaling cluster (cluster 1) within COVID-19 samples, we further compared the expression of type I–specific (ISG-I) and type II–specific IFN-stimulated genes (ISG-II) between COVID-19^+^ cases and healthy controls and within COVID-19^+^ cases of varying severity. To differentiate ISG-I and ISG-II, we compared healthy donor PBMCs stimulated with recombinant IFN-β or IFN-γ from an independent single-cell RNA sequencing (scRNA-seq) dataset to identify genes specifically up-regulated by either IFN (Fig. 3B). In myeloid cells (cM, ncM, cDC, and pDC), the average expression of ISG-I, and to a lesser extent ISG-II, in critical cases on day 0 of hospitalization was significantly lower compared to moderate and severe cases [ISG-I: log_2_FC = −0.51 to −0.82, *P* = 8.6 × 10^−4^ to 6.9 × 10^−3^; ISG-II (cM only): log_2_FC = −0.46, *P* = 3.1 × 10^−3^] (Fig. 3C and fig. S5, B and C). Furthermore, in DCs (cDC and pDC), ISG-I expression for the four critical COVID-19^+^ cases with anti–IFN-α2 autoantibodies was significantly lower than that for critical cases without autoantibodies [ISG-I: log_2_FC = −0.75 to −1.05, *P* = 2.0 × 10^−2^ to 2.8 × 10^−2^; ISG-II (pDC only): log_2_FC = −0.92, *P* = 2.1 × 10^−3^; Fig. 3C]. Through the disease course, average expression of ISG-I in moderate cases was high at the time of hospitalization but quickly diminished, whereas in critical cases, especially those with anti–IFN-α2 autoantibodies, average expression of ISG-Is remained low [linear mixed model (LMM); moderate: slope = −4.8 × 10^−3^, *P* < 0.05; other groups, *P* > 0.05; Fig. 3D]. This deficient ISG-I response in critical versus moderate-severe cases independent of days since symptom onset is consistent with previous literature (*30*). These findings suggest that there may be a shared causal mechanism of critical disease in patients with and without autoantibodies to type I IFNs.

### ISG-I deficiency is associated with elevated surface expression of leukocyte-associated Ig-like receptor 1 in myeloid cells

**Fig. 4.**
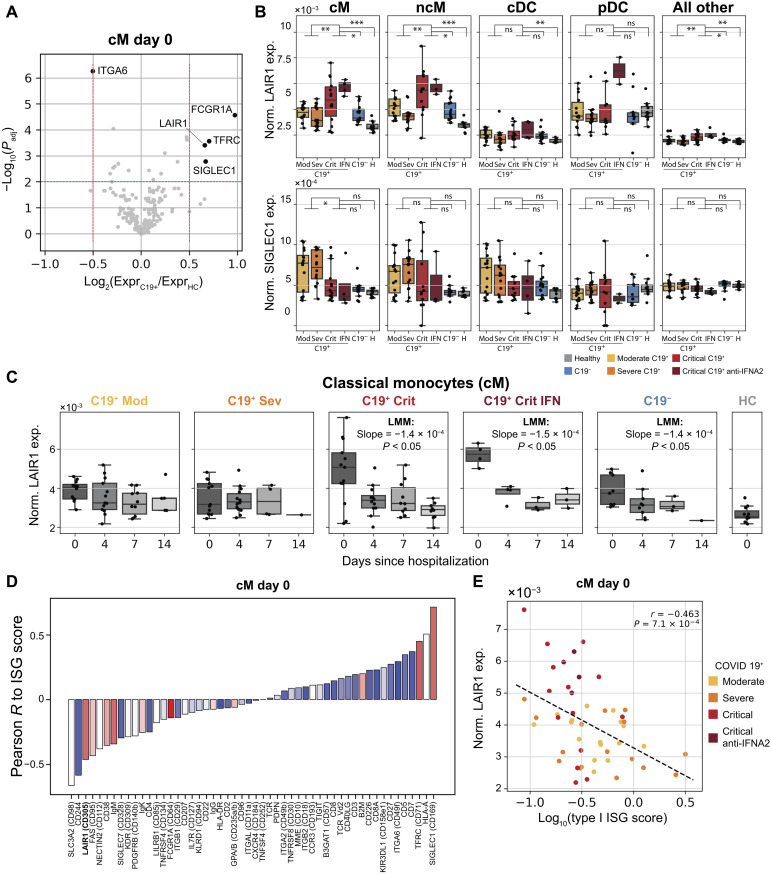
Surface protein abundance changes are observed in leukocyte subsets of patients with critical COVID-19. (**A**) Volcano plot of log fold change between COVID-19^+^ and healthy controls (*x* axis) versus −log10(*P* value) (*y* axis) in cM cells. Proteins that are significantly (FDR < 0.05) differentially abundant and have a log_2_(fold change) > 0.5 are highlighted. (**B**) Normalized (Norm.) LAIR1 and SIGLEC1 surface expression (exp.; *y* axis) at day 0 across eight cell types separated by case control status, severity, and presence of anti–IFN-α2 antibodies (*x* axis). Box plots show median and 25th and 75th percentile. (**C**) Normalized LAIR1 surface expression (*y* axis) in cMs over the course of disease for healthy controls, COVID-19^−^ controls, and COVID-19^+^ cases. COVID-19^−^ controls and COVID-19^+^ cases are separate by severity and the presence of anti–IFN-α2 antibodies. Insets show significant results of LMM testing for changes over time. (**D**) The bar plot shows correlation between the surface expression of 52 statistically significantly proteins to ISG-I score in cMs at day 0. Proteins are colored by their log_2_(fold change) expression between COVID-19^+^ cases and healthy controls. Red indicates higher expression in COVID-19^+^ cases. Blue indicates lower expression in COVID-19^+^ cases. (**E**) Scatter plot of normalized LAIR1 expression (*y* axis) versus the ISG-I score (*x* axis) for COVID-19^+^ cases colored by severity and anti–IFN-α2 status. *P < 0.05, **P < 0.01, and ***P < 0.001.

We next sought to identify changes in the expression of surface proteins in patients with COVID-19 that may be correlated with ISG-I expression. The correlation of surface protein and transcript abundance varied across the 189 targeted genes, with lineage-specific surface markers exhibiting the highest correlation (fig. S6A). Comparing COVID-19^+^ cases at day 0 of hospitalization to healthy controls for each cell type separately, we identified 5 of 189 differentially expressed surface proteins in cMs and an additional 14 of 189 in other cell types (|log_2_FC| > 0.5, FDR < 0.05; Fig. 4A and fig. S6B). Of the five proteins differentially expressed in cMs, four [transferrin receptor (TFRC), sialic acid binding Ig-like lectin 1 (SIGLEC1), Fc fragment of IgG receptor 1a (FCGR1A), and leukocyte-associated Ig-like receptor 1 (LAIR1)] were higher expressed in COVID-19^+^ cases. SIGLEC1 is a known up-regulated ISG (*31*) whose pattern of surface expression is consistent with the expression of ISG-I present in moderate and severe cases (Figs. 3C and 4B). In contrast, LAIR1, a collagen receptor (*32*, *33*) and a type 1 Ig containing two immunoreceptor tyrosine-based inhibitory motifs (*34*), was most differentially up-regulated in myeloid cell types of critical COVID-19^+^ cases compared to healthy controls (cM, ncM, and cDC: log_2_FC = 0.51 to 0.97, *P*_adjusted_ = 4.8 × 10^−3^ to 3.9 × 10^−5^) and compared to moderate-severe COVID-19^+^ cases (cM and ncM: log_2_FC = 0.47 to 0.50, *P*_adjusted_ = 1.2 × 10^−2^ to 7.9 × 10^−3^; Fig. 4B and fig. S6B). LAIR1 expression in cMs from critical COVID-19^+^ cases was high early in the disease course and significantly decreased over time in those patients (LMM; COVID-19^+^ critical: slope = −1.3 × 10^−4^, *P* < 0.05; COVID-19^+^ critical anti-IFN: slope = −1.5 × 10^−4^, *P* < 0.05; Fig. 4C and fig. S6C). *LAIR1* transcripts were also differentially expressed by cM, ncM, cDC, and NK cells in COVID-19^+^ cases when compared to healthy controls (log_2_FC = 0.65 to 1.06, FDR = 5.0 × 10^−6^ to 3.0 × 10^−3^; other cell types: log_2_FC = −1.2 × 10^−2^ to 1.6 × 10^−2^, FDR = 0.64 to 0.93), and the transcript and protein abundances were correlated (mean across all cell types: Pearson *R* = 0.89, *P* = 2.3 × 10^−4^). In COVID-19^+^ cases at day 0, the expression of surface LAIR1 was inversely correlated with expression of ISG-Is in cMs (Pearson *R* = −0.47, *P* < 0.01) and ncMs (Pearson *R* = −0.41, *P* < 0.01) (Fig. 4, D and E, and fig. S6D). Furthermore, LAIR1 surface protein expression for 40 samples was significantly correlated with those obtained from flow cytometry (Pearson *R* = 0.43, *P* = 5.5 × 10^−3^) (fig. S6E). Unlike known IFN-repressed surface proteins [such as CD244 (*33*)] that were also inversely correlated with the ISG-I score (Fig. 4D), LAIR1 is not expressed in healthy samples, suggesting that it is not an IFN-repressed gene. These results demonstrate high surface expression of LAIR1 in myeloid cells to be a biomarker predictive of deficient type I–specific IFN response.

## DISCUSSION

The marked clinical heterogeneity over the course of SARS-CoV-2 infection, ranging from asymptomatic to lethal, is a key observation and defining feature of the COVID-19 pandemic. It is important to understand what causes life-threatening COVID-19 pneumonia in a minority of infected individuals. Recent work has suggested that preexisting autoimmunity against type I IFN can underlie critical COVID-19 pneumonia in greater than 10% of cases (*15*). Here, we have confirmed that neutralizing autoantibodies to type I IFNs are present in patients with severe to critical COVID-19 from two independent cohorts, showing a combined prevalence of about 9%. Both the COVID-19^+^ and asymptomatic cohorts studied here had substantial Hispanic representation, a population that has not been previously studied at scale. The presence of anti–type I IFN autoantibodies in this population indicates that this phenomenon may be widely conserved across a diversity of ancestries. In terms of age and gender, the majority of autoantibody-positive severe to critical COVID-19 cases were male and over 55 years of age, consistent with previous reports (*15*). We observed roughly equal numbers of autoantibody-positive males and females in the general population of San Francisco, as assessed through our community survey. Further study will be required to determine whether differences exist between the autoantibodies in male versus female individuals before SARS-CoV-2 infection that could partially explain the downstream skewing of hospitalized patients toward male gender, such as differential neutralization ability or additional accompanying risk factors.

In four patients with critical COVID-19 and tested positive for anti–type I IFN autoantibodies, longitudinal analysis determined that these autoantibodies were present from the earliest time point in their clinical course (collected within 4 to 13 days from the start of their first disease symptoms). Given the time required for a detectable, stable humoral immune response to form (2 to 3 weeks) (*36*), our data suggest that autoantibodies predate infection with SARS-CoV-2. Consistent with this, our survey of a community cohort in the San Francisco Mission District also revealed a subset of presumed uninfected individuals who were anti–type I IFN antibody positive (0.3%), suggesting that there are individuals who may be at higher risk for critical COVID-19 due to these preexisting autoantibodies, including both males and females across a broad range of ages. Moreover, in a community-based population study, we did not detect these autoantibodies in 154 patients with asymptomatic or ambulatory infection with SARS-CoV-2 (compared to 13 of 3821 uninfected individuals) or in CCP donor samples, suggesting that the penetrance of severe to critical COVID-19 in infected individuals with autoantibodies is potentially complete.

In addition to validating the presence of anti–type I IFN autoantibodies in patients with severe and critical COVID-19, we further showed, using single-cell transcriptomic analysis, that these antibodies are associated with impaired ISG-I response in several distinct myeloid populations. Although other similar high-dimensional immune profiling studies have found evidence of impaired ISG responses in monocytes (*37*, *38*) and neutrophils (*25*), we have now provided additional specificity and a potential mechanism of how this may unfold in a subset of individuals. We also found impaired myeloid ISG-I expression in additional individuals with critical disease without detectable anti–type I IFN autoantibodies. This observation suggests that impaired type I IFN immunity is a shared mechanism of more severe forms of the disease in patients with and without autoantibodies to type I IFNs (*13*). It is possible that patients without detectable autoantibodies may have had lower titers of autoantibodies, autoantibodies that neutralized lower amounts of type I IFNs, or autoantibodies that were undetectable because they were bound to type I IFNs. Alternatively, these patients may carry inborn errors of the production and amplification of type I IFNs, as recently shown in other patients (*13*) or autoantibodies against other cytokines or proteins in type I IFN response (*39*). It is also possible that other antibody-mediated mechanisms may exist that are independent of the direct binding to IFNs (*25*). Genetic and immunological studies are underway in our cohort of patients. These findings, along with the observation of high ISG-I expression in moderate patients early during the disease course that quickly diminishes, further suggest that impaired type I IFN immunity during the first hours and days of infection may account for the protracted disease course, including pulmonary and systemic inflammation. A two-step model of life-threatening COVID-19 is emerging, with defective type I IFN intrinsic immunity in the first days of infection resulting in viral spread, in turn unleashing leukocyte-mediated excessive inflammation in the lungs and other infected organs during the second week of infection (*12*).

Our analysis of 189 cell surface proteins identified the expression of LAIR1 in cMs to be elevated in patients with COVID-19 and correlated with the impaired ISG-I response. LAIR1 is an inhibitory surface protein first described to be expressed in lymphocytes and involved in inhibiting NK-mediated cell lysis and effector T cell cytotoxicity upon FcR-mediated cross-linking (*34*, *40*, *41*). More recently, it has also been shown in monocytes and pDCs that LAIR1 cross-linking or binding to cognate ligands collagen and C1Q can inhibit the production of IFNα in response to Toll-like receptor ligands in healthy controls and patients with systemic lupus erythematosus (*42*, *43*). LAIR1 expression is highest in cMs at the time of initial hospitalization and decreases rapidly by day 4 among a subset of critical patients, including the four with anti–type I IFN autoantibodies. In a recent study, a large array of autoantibodies were characterized in patients with COVID-19, among which autoantibodies against LAIR1 that were found to be highly specific to severe-to-critical COVID-19 (*39*). Whether LAIR1 plays a causal role in deficient type I IFN response would require further investigation. Nevertheless, the ability to use a highly cell type–specific surface protein to predict impaired type I IFN response in patients with critical COVID-19 early during disease provides an important biomarker.

Our study has several limitations. First, we do not formally have age-, sex-, or ancestry-matched individuals presenting with different disease severity or in our control groups for the single-cell analysis. However, we did not detect associations to age or sex on cell composition or ISG-I expression for critical patients with or without autoantibodies. Second, there is a slightly longer delay between symptom onset and hospitalization with increasing disease severity (moderate: 2 to 11 days, 6.5 days average; severe: 2 to 16 days, 10.4 days average; critical: 6 to 36 days, 13.2 days average) and a bias toward specific patients with comorbidities or more severe disease at later time points. Nevertheless, these differences in the dynamics of the disease course do not fully explain the dynamics of ISG-I expression because the responses in moderate and severe patients never reach the low expression observed in critical patients even after 14 days of hospitalization, something that is in line with previous literature (*30*). Third, because we only had sequencing data from four anti–IFN-α2 autoantibody-positive patients, there was limited power to compare critical cases with and without anti–IFN-α2 autoantibodies.

In conclusion, our findings have several important implications for the ongoing COVID-19 pandemic and our understanding of patients with a critical clinical course. First, our results show that an impaired ISG-I response early in the disease course in multiple immune populations is associated with autoantibodies to type I IFNs, providing a glimpse into the immune dysregulation present in patients with a severe clinical course. In this regard, it is critical to be able to identify patients with an impaired ISG-I response early during disease course; a combination of the highly specific assays for autoantibodies against type I IFNs and biomarkers for deficient ISGs, such as LAIR1, could quickly allow triaging of patients during initial hospitalization. Second, treatment strategies with IFN-β might be particularly valuable for those with preexisting antibodies to type I IFNs because these appear to neutralize only IFN-α or IFN-ω (*16*). The large immunological differences of severe to critical patients in the earliest time points additionally suggest that identification and treatment would likely need to happen early in the disease course. Third, we found that autoantibodies to type I IFNs in critical COVID-19 cases were present at the time of their presentation and precede the development of antibodies to SARS-CoV-2. This, along with the presence of healthy autoantibody-positive individuals in the community (*16*), suggests that anti–type I IFN autoantibodies predate infection and that there exists an at-risk group for severe to critical disease in the general population. Going forward, strategic efforts to identify this high-risk population early in the disease course could have substantial impact on improving clinical outcomes including mortality rates, and identifying these individuals before infection could have a major impact on preventive measures.

## MATERIALS AND METHODS

### Study design

This study was designed to identify immunological features in the blood from hospitalized patients presenting with COVID-19 symptoms associated with disease status and severity. For this purpose, PBMCs and serum samples were collected from participants enrolled in the COMET cohort, which partially includes patients recruited to the Immunophenotyping Assessment in a COVID-19 Cohort (IMPACC) study (data file S4). The materials collected from each of the participants in this study were coded by donor ID numbers such that the experimentalist could not define the disease status of each participant. Hence, in each experiment, we randomized the processing of PBMCs from 22 to 25 participants, including 16 to 23 patients and 2 to 8 healthy individuals. All patients hospitalized for symptomatic COVID-19 infection at both the tertiary care center and the safety-net county hospital associated with the University of California San Francisco (UCSF) were eligible to participate in the COMET cohort study. Biospecimens may have been collected under an Institutional Review Board (IRB)–approved initial waiver of consent with subsequent attempts to consent surrogates and study participants for full study participation. We selected samples from 69 of 101 of participants enrolled in COMET between 8 April 2020 and 20 June 2020 (Table 2 and data file S1), which resulted in at least 15 to 20 donors per COVID-19 severity group. According to previous publications of similar cohort sizes, our study should have sufficient power to detect COVID-19–associated cell type compositional and gene expression changes of similar effect sizes as described (*29*, *38*). The healthy anonymized donors were chosen within the age range of patients with COVID-19. Sample selection was prioritized in the patients that were hospitalized with longitudinal blood collections, and therefore, PBMCs were available across a 14-day period. This study is approved by the IRB of the UCSF Human Research Protection Program (IRB no. 20-30497).

A total of 231 patients with COVID-19 and 96 healthy controls were enrolled in the ZSFG cohort (Table 3 and data file S2). All ZSFG patients were collected between 1 March 2020 and 21 July 2020 and had positive results by SARS-CoV-2 reverse transcription PCR (RT-PCR) in nasopharyngeal swabs. Clinical data were extracted from electronic health records and included demographic information, major comorbidities, patient-reported symptom onset date, symptoms, and indicators of disease severity. Healthy, pre–COVID-19 control plasma samples were obtained as deidentified samples from the New York Blood Center. These samples were part of retention tubes collected at the time of blood donations from volunteer donors who provided informed consent for their samples to be used for research.

Details of the community-based cohort including 4041 participants were previously described by Chamie *et al.* (*27*) (Table 4 and data file S3). The four APS-1 patients in the study were collected at the NIH under protocol #11-I-0187 and were previously published by Ferre *et al.* (*44*, *45*). CCPs were collected in the Vitalant system following U.S. Food and Drug Administration (FDA) guidance for donor eligibility. These criteria evolved throughout the study period because of testing availability and evolution of the pandemic in the United States. Evidence of COVID-19 was required in the form of a documented positive SARS-CoV-2 molecular or serologic test and complete resolution of symptoms initially at least 14 days before donation, but then, a minimum of 28 days was implemented. All CCP donors were also required to meet traditional allogeneic blood donor criteria. At the time of plasma collection, donors consented to use of deidentified donor information and test results for research purposes. All CCPs were tested for SARS-CoV-2 total Ig antibody using the Ortho VITROS CoV2T assay at our central testing laboratory (Creative Testing Solutions). CCP qualification requires the signal-to-cutoff (S:CO) ratio of this test to be at least 1.0. Retention samples of serum and plasma for all donations are archived at the Vitalant Research Institute Denver. Plasma samples are from 175 unique CCP donors, where some had repeated donations, for a total of 281 samples. These samples were selected solely on the Ortho VITROS CoV2T assay results to represent the entire range of high to low S:CO ratio. Collections were from across the U.S. Vitalant system from 8 April 2020 to 1 September 2020.

### Isolation and preparation of PBMCs for scRNA-seq

Whole blood from 80 donors was drawn into plastic EDTA Vacutainer blood collection tubes (Becton, Dickinson and Company) at the time of hospital admission (D0) and 4 (D4), 7 (D7), and 14 (D14) days later. Of these donors, 69 were patients with high clinical suspicion of COVID-19 infection that were admitted at UCSF or Zuckerberg San Francisco General Hospital and Trauma Center (ZSFG) and 11 were healthy donors. COVID-19 status was assessed for all 69 patients by RT-PCR tests of nasal swab samples and confirmed that 15 patients were COVID-19 negative, whereas 54 patients were COVID-19 positive. During the hospitalization, the severity of each COVID-19–positive patient was assessed using the NIH COVID-19 severity scale (Table 1) (*26*). For all analyses, we categorized patients on the basis of their severity at time of hospital admission (D0).

PBMCs were isolated at room temperature using SepMate PBMC Isolation Tubes (STEMCELL Technologies) by the UCSF Biospecimen Resource Program. Briefly, 7.5 ml of whole blood was centrifuged at 1000 relative centrifugal force (rcf) for 10 min with swinging-out rotor and brake off to separate 3.5 ml of plasma. The remaining blood was diluted with 8 ml of Dulbecco’s phosphate-buffered saline (DPBS) and slowly added to a SepMate-50 tube prefilled with 15 ml of Lymphoprep (STEMCELL Technologies). The tube was then centrifuged at 800 rcf for 20 min with brakes off. After centrifugation, the top layer including the buffy coat was gently and quickly poured into a 50-ml Falcon tube to centrifuge at 400 rcf for 10 min with brake on. The pellet was washed twice each time with 20 ml of EasySep buffer (STEMCELL Technologies) followed by centrifugation at 400 rcf for 10 min with brakes on. Washed PBMCs were counted and resuspended at 5 × 10^6^ cells/ml in cold freezing media (fetal bovine serum with 10% dimethyl sulfoxide solution). Cells were aliquoted into cryovials at 1 × 10^6^ to 5 × 10^6^ cells per vial and transferred in a Mr. Frosty (Nalgene) to the −80°C freezer for 24 hours before cryopreservation in liquid nitrogen.

### Single-cell multimodal immunophenotyping

Multiplexed single-cell sequencing was performed following a previously published protocol (*46*) and manufacturer’s user guide (Document CG000186 Rev D, 10X Genomics). The complete protocol is available on protocols.io (www.protocols.io/view/10x-citeseq-protocol-covid-19-patient-samples-tetr-bqnqmvdw). In each experiment, PBMCs from 22 to 25 participants were used, including 16 to 23 patients and 2 to 8 healthy individuals. Longitudinal samples of the same patient were used in different experiments to allow genetic demultiplexing. Each experiment used samples collected at different longitudinal time points to prevent experimental conditions from coinciding with potential batch effects.

Briefly, PBMCs were thawed in a 37°C water bath for 30 s and washed with 5 ml of complete RPMI medium followed by centrifugation at 350 rcf for 5 min at room temperature. Cell counts and viability were determined using a Vi-CELL XR automated cell counter (Beckman Coulter Life Sciences). An equal number of cells were aliquoted from each sample to create a pool of 1.5 × 10^6^ cells with an average viability of 85% or higher. Pooled PBMCs were blocked with Human TruStain FcX (BioLegend) in cell staining buffer (BioLegend) for 10 min on ice, followed by staining with a customized TotalSeq-C human cocktail for 45 min on ice (data file S5). Cells were washed three times, resuspended in PBS with 0.04% bovine serum albumin, filtered through a 40-μm filter, and counted with Countess II Automated Cell Counter (Thermo Fisher Scientific). Single-cell suspensions were loaded into a Chromium Single Cell Chip A for single cell encapsulation using the 10X Chromium Controller according to the manufacturer’s user guide (Document CG000186 Rev D, 10X Genomics) and as previously described (*47*). In each experiment, the pooled cells of 22 to 25 participants were loaded into four to six individual lanes aiming for 7 × 10^4^ loaded cells per lane.

### Single-cell library preparation and sequencing

Single-cell libraries were constructed following the manufacturer’s user guide (Document CG000186 Rev D). Complementary DNA (cDNA) libraries were generated using the Chromium Single Cell 5′ Library & Gel Bead Kit and i7 Multiplex Kit. Surface protein Feature Barcode libraries were generated with Chromium Single Cell 5′ Feature Barcode Library Kit and i7 Multiplex Kit N, Set A. In total, libraries generated from 971,550 cells were PE150-sequenced at the Chan Zuckerberg Biohub on 18 lanes of an Illumina NovaSeq 6000 sequencer using a NovaSeq 6000 S4 Reagent Kit v1.

### Genotyping, sample demultiplexing, and doublet detection

To assign cells to donors of origin in our multiplexed design, we used the genetic demultiplexing tools Freemuxlet and vcf-match-sample-ids, each a part of the Popscle suite of statistical genetics tools (https://github.com/statgen/popscle). Freemuxlet leverages the single-nucleotide polymorphisms (SNPs) present in transcripts and performs unsupervised clustering on the droplet barcodes to assign each to a nameless donor or assign them as doublets between genetically distinct nameless donors. The algorithm takes in a list of candidate loci throughout the genome at which to scan for SNPs and returns droplet barcodes with donor assignments and a set of observed variants per donor. These sets of variants are then matched using genotypic similarity to those from an orthogonal bulk RNA-seq assay, done on an individual basis, to determine which donor is which patient. Once nameless donors are matched to uniquely identifiable patients, droplet data are then joined with the other clinical covariates available for the patients, including age, sex, race, and disease status. Freemuxlet was run on each of the nine droplet reaction runs separately, using a list of exonic SNPs that were expected to be found in the 5′-end scRNA-seq data and that have a minor allele frequency >0.05, based on data from the 1000 genomes project (*47*).

### Bulk RNA sequencing

Bulk RNA-seq data were generated to extract genotype information so that single cells could be demultiplexed and matched to single donors. For each donor, RNA was extracted from PBMCs using the Quick-RNA MagBead Kit (Zymo Research) on a KingFisher Flex system (Thermo Fisher Scientific) according to the manufacturer’s protocol. RNA integrity was measured with the Fragment Analyzer (Agilent), and library generation was continued when integrity was at least 6. Total RNA-seq libraries were depleted from ribosomal and hemoglobin RNAs and generated using FastSelect (Qiagen) and Universal Plus mRNA-Seq with NuQuant (Tecan) reagents. Pooled libraries were PE100-sequenced on a HiSeq 4000 or PE150-sequenced on an Illumina NovaSeq 6000 S4 flow cell at the Chan Zuckerberg Biohub.

### Single-cell epitope and RNA-seq preprocessing and alignment

CellRanger v3.1.0 (run 1 to 7, cDNA library generation of run 6 failed) or v4.0.0 (run 8 to 10) software with the default settings was used to demultiplex the sequencing data and generate FASTQ files (Cellranger mkfastq), align the sequencing reads to the hg38 reference genome, and generate a unique molecular identifier (UMI)–filtered gene and protein expression count matrix for each lane (Cellranger count for scRNA-seq and CITE-seq data). Count matrices were then concatenated across all 50 lanes to generate two matrices: one mRNA matrix with 971,550 cells and 36,601 genes, and one surface protein matrix with 971,550 cells and 189 proteins.

### Single-cell epitope and RNA-seq processing and quality control

Resulting gene and protein expression count matrices were further processed in the Python package Scanpy v1.5.1 (*49*). Processing of the concatenated mRNA count matrix was done using a two-step process. We have found empirically that traditional workflows for the processing of droplet-based RNA-seq data for PBMCs can sometimes create unwanted effects in the downstream end points. In particular, filtering of cells with a high percentage of mitochondrial cells may create visual artifacts in UMAP projections, and filtering of the matrix to only a few hundred highly variable genes and reducing the memory footprint of the data can sometimes lead to spurious clustering of cells based on only a few genes. Our iterative process yields a UMAP projection that captures all available heterogeneity with minimal filtering in the first iteration, then removes nontarget cells, and corrects for nonbiological signal in the second iteration. By doing this, we use a relatively large number of components to inform projections and clustering but observe that the outputs in our dataset match the known biology better (such as proximity of similar cell types and states in UMAP space) and yield higher-confidence annotations.

In the first step, the mRNA matrix was filtered to remove doublet droplets, as annotated by Freemuxlet, and very lowly expressed genes with less than 100 UMIs across the nine runs. The matrix was then normalized to yield a constant UMI sum per cell and log-transformed. Matrix values were scaled to yield a mean of zero and SD of 1 per gene. Principal components analysis (PCA) was performed, followed by nearest neighbors, UMAP projection, and Leiden clustering, using an input of the 150 principal components with the highest variance explained and otherwise default Scanpy parameters. At this stage, Leiden clustering resolution was adjusted and restricted to certain clusters to separate out clusters of cells that projected into similar UMAP space. These clusters were subsequently annotated to mark those with a high percentage of mitochondrial content, which typically represent dead or dying cells, as well as mark clusters with high expression of hemoglobin and platelet factor 4, representing the nontarget cell types of red blood cells and platelets. At this stage, we also observed that there were prominent batch effects in UMAP space that required correction.

In the second iteration, nontarget cell types marked in the first step were removed before processing. Then, the same processing was followed starting from the raw data, with the exception that ComBat batch correction (*50*) was performed (to correct for the “run” covariate) after scaling and before performing PCA. Last, further filtration of a relatively small number of cells with high expression of platelet, red blood cell, and mitochondrial genes was performed, as well as removal of donors that declined study participation. After processing, 600,929 cells and 18,262 genes remained in the mRNA matrix.

The surface protein matrix was filtered to the cells found in the mRNA matrix. One protein was removed from the matrix, as it appeared to have very low counts relative to the other surface proteins. The remaining proteins were then normalized using the centered-log ratio (CLR), computed for each gene independently. The CLR has typically been used for CITE-seq data with the recognition that antibody counts are typically not zero-inflated and flow cytometry–like Gaussian distributions are achievable when treating the data as compositional. However, we recognized that the CLR-normalized distributions were affected by a relatively small number of cells that had extremely high or low expression, skewing the visualization of the Gaussian mixture distributions. To remedy this effect, we identified boundary values of the distributions for each gene using a bin height threshold when values were plotted on a histogram, clipped the values to these boundaries, and scaled the remaining values between 0 and 1.

### Cell type classification

After processing, Leiden clustering was adjusted to match the clustering of cells projected into UMAP space. Cell type annotation was performed at three levels of granularity based on known marker gene and protein expression, as well as differentially expressed genes between clusters using a Wilcoxon rank-sum test. At the lowest level of granularity, we identified 11 cell types corresponding to the known major cell types present in PBMCs: CD4T, CD8T, γδT, cMs, ncMs, NK, B, PBs, cDC, pDC, and Progen cells. At the next level of granularity, we separate out memory, naïve, and proliferating subtypes in the lymphocytes; two different subtypes known in each of the NK cells and conventional DCs; regulatory T cells from the CD4T group; mucosal-associated invariant T cells from the CD8T group; subpopulations of lineage-committed progenitor cells; and a subpopulation of B cells that seemed to be committed to the PB lineage. At the highest level of granularity, we further separate out a CD8^+^ effector memory T cell population, an NK population with CD3 transcript expression, early and late proliferating subpopulations in the lymphocytes, and a few subpopulations that were either donor specific (patient 1002 in the B cells and patient 1006 in the monocytes) or run specific [such as cells from run 3, which exhibited some processing issues and for which ComBat (*50*) was unable to correct].

### Differential proportion and expression analysis

Differences in cell type proportions were assessed in COVID-19^+^ versus COVID-19^−^ cases or healthy controls by aggregating cell type observations per COVID-19 status at time point D0. In addition, in the COVID-19^+^ cases for which all four time points were available, cell type proportion changes were assessed over time. Differences in gene expression were determined for each of the myeloid cell types between COVID-19^+^ cases at D0, D4, D7, or D14 versus healthy controls. To assess whether these changes are specific for COVID-19^+^ cases or are a more general phenomenon due to acute respiratory distress syndrome, we also compared the up-regulated genes with the COVID-19^−^ cases. Differential expression analysis was performed per run on the raw gene expression matrix using Memento v0.0.4 (doi: 10.5281/zenodo.5172943), after which results were meta-analyzed over all runs. Genes were prefiltered on the basis of a minimum raw mean expression of 0.07 within at least 90% of both comparison group.

### Differential expression heatmaps

Heatmaps show the pseudobulked, *z*-scored expression values of the donors present at each time point for the top significantly up-regulated genes. To generate the heatmaps, cells were first subsetted from the larger mRNA matrix to only those of a given cell type. Counts were pseudobulked across all genes by patient present at each time point, yielding a single gene-by-sample matrix, with 179 unique donor–time point samples. The genes in this matrix were subsetted to the union of the top 150 genes with the highest differential expression coefficient at each time point, using the one-dimensional memento results that tested gene counts in COVID-19^+^ cases versus healthy controls. Genes were further filtered to remove those that had high variance in healthy controls (SD > 0.5) because these were enriched for what seemed to be a nonbiological signal (such as ribosome-associated genes). The matrix, now with 204 genes, was then z-scored and separated by time point to four matrices, with healthy samples being distributed to each matrix.

Ordering of the rows and columns were computed such that they would be consistent among the heatmaps. Genes were clustered by *k* means using only the values of the day 0 time point, with *k* = 6 chosen by the “elbow” point of the graph plotting distortion (using a sum of square errors cost function) with increasing numbers of clusters. Columns of each heatmap were determined by taking the 80 columns across all heatmaps that had the earliest time point for each donor, subsetting according to their disease status (healthy, COVID-19^−^, and COVID-19^+^), and then hierarchically clustering within each of those groups. With this ordering, each donor then had a unique position along the horizontal axis, which was then applied to all the heatmaps, omitting those samples that were absent from a given time point. GSEA was done using the GOATOOLS Python package (*51*), filtering to terms with at least two associated genes.

### ISG score method

An orthogonal scRNA-seq dataset containing PBMCs stimulated with IFN-β and IFN-γ was used to identify the specific and shared type I and type II ISGs in the cMs (Gene Expression Omnibus accession number GSE181897). The gene list was used to calculate a type I, type II, and shared ISG score based on the average gene expression count of the unique or shared type I and type II ISGs. These ISG scores were calculated for each unique combination of cell type, donor, and time point. Subsequently, ISG scores were averaged over each of the disease categories (COVID-19^+^ moderate-severe, COVID-19^−^ moderate-severe, healthy control) and then log_2_-transformed.

### Flow cytometry validation of elevated surface LAIR1 in cMs for patients with COVID-19

Frozen PBMCs from patients with available samples (8 critical, 9 moderate, 8 severe, 10 negative controls, and 5 healthy controls) were thawed as previously mentioned. Each sample (5 × 10^5^ cells per donor) was then resuspended to 75 μl of cell staining buffer and aliquoted to a 96-well U-bottom plate (Genesee Scientific catalog no. 25-221) before blocking with 5 μl of TruStain FcX for 10 min on ice. Cells were then stained with a cocktail of CD14–Brilliant Violet 421 (BioLegend, clone M5E2, catalog no. 301830), CD16–Alexa Fluor (AF) 488 (BioLegend, clone 3G8, catalog no. 302019), SIGLEC1-phycoerythrin (BioLegend, clone 7-239, catalog no. 346004), and LAIR1-AF647 (BioLegend, clone NKTA255, catalog no. 342802) antibodies in a total volume of 100 μl (5 μl per antibody) for 30 min on ice in the dark. Cells were washed twice in 200 μl of cell staining buffer before resuspension in 300 μl of PBS. Cells were stained with propidium iodide (PI; final 1 μg/ml) before flow analysis on an LSR II (UCSF Parnassus Flow Cytometry Core). Analyzed cells were gated for singlets, live (PI^−^), and CD14^+^CD16^−^ before mean fluorescence intensity (MFI) calculations.

### SARS-CoV-2 detection by clinical RT quantitative PCR

Viral titers were quantified in a subset of the COVID-19^+^ patients in the UCSF CLIAHUB Clinical Microbiology Laboratory. For this, RNA was extracted from tracheal aspirate and nasopharyngeal swabs and used for RT-qPCR (quantitative PCR) as previously described (*52*). In short, viral titers were assessed using primers targeting the SARS-CoV-2 N gene (Ct1) and E gene (Ct2) and human ribonuclease (RNase) P gene (Ct_host, positive control). The Ct value of the viral N or E gene was subtracted from the human RNase P gene (ΔCt: ΔCt1 and ΔCt2) (data file S9), and number signs were reversed to obtain a measurement for normalized viral RNA abundance. As there was an almost perfect correlation between ΔCt1 and ΔCt2 values (Pearson *R* = 0.99, *P* = 1.9 x 10^−283^) and ΔCt2 had the least missing values, viral RNA abundance is represented by the ΔCt2 values. ΔCt2 values as measured in the tracheal aspirate and nasopharyngeal swab samples were linked to the scRNA-seq PBMC data of the same donor and the closest possible time point (up to 2 days apart).

### RLBA for anti–IFN-α2 autoantibody detection

A DNA plasmid containing full-length cDNA sequence with a Flag-Myc tag (OriGene, #RC221091) was verified by Sanger sequencing and used as template in T7-promoter–based in vitro transcription/translation reactions (Promega, #L1170) using [S35]-methionine (PerkinElmer, #NEG709A). IFN-α2 protein was column-purified using NAP-5 columns (GE Healthcare, #17-0853-01); incubated with 2.5 μl of serum, 2.5 μl of plasma, or 1 μl of anti-myc–positive control antibody (Cell Signaling Technology, #2272); and immunoprecipitated with Sephadex protein A/G beads (4:1 ratio; Sigma-Aldrich, #GE17-5280-02 and #GE17-0618-05) in 96-well polyvinylidene difluoride filtration plates (Corning, #EK-680860). The radioactive counts [counts per minute (cpm)] of immunoprecipitated protein were quantified using a 96-well MicroBeta TriLux liquid scintillation plate reader (PerkinElmer). Antibody index for each sample was calculated as follows: (sample cpm value – mean blank cpm value)/(positive control antibody cpm value – mean blank cpm value). For the COVID-19 patient and CCP cohorts, a positive signal was defined as greater than 6 SDs above the mean of pre–COVID-19 blood bank noninflammatory controls. For the large asymptomatic San Francisco community population cohort, a positive signal was defined as having a *z* score greater than 3.3 (*P* = 0.0005) relative to the whole cohort.

### Luciferase reporter assays

The blocking activity of anti–IFN-α and anti–IFN-ω autoantibodies was determined by assessing a reporter luciferase activity. Briefly, human embryonic kidney (HEK) 293T cells were transfected with the firefly luciferase plasmids under the control human *ISRE* promoters in the pGL4.45 backbone and a constitutively expressing *Renilla* luciferase plasmid for normalization (pRL-SV40). Cells were transfected in the presence of the X-tremeGENE 9 transfection reagent (Sigma-Aldrich) for 36 hours. Dulbecco’s modified Eagle’s medium (Thermo Fisher Scientific) was supplemented with 10% healthy control or patient serum or plasma and was either stimulated with IFN-α or IFN-ω (10 ng/ml) or left unstimulated for 16 hours at 37°C. Each sample was tested once. Last, luciferase expression was measured with the Dual-Glo reagent according to the manufacturer’s protocol (Promega). Firefly luciferase values were normalized against *Renilla* luciferase values, and fold induction was calculated relative to controls transfected with empty plasmids.

### Statistical analysis

Differential proportion analysis was performed using a permutation-based approach that compares observed cell type proportion differences with those calculated from a null distribution that is generated by randomly shuffling cell type labels (100,000 permutations) for a fraction (*w* = 0.1) of the total cells, as described previously (*53*). Resulting *P* values were corrected for multiple testing using Holm’s correction, after which an adjusted *P* value of <0.05 was considered significant. For the differential expression analysis, an FDR of <0.05 was used to determine statistical significance. A Welch’s *t* test was performed to compare the ISG score between COVID-19^+^ patients and healthy controls. Significance was defined as Bonferroni-adjusted *P* value < 0.05. To detect changes in ISG-I score or LAIR1 protein expression over time, samples were first subsetted to COVID-19^+^ and COVID-19^−^ patients for whom we had more than one time point (54 of 69). We then used an LMM for each group using the statsmodels Python package, with formula “<measurement> ~ time_point,” where <measurement> was either type I score or LAIR1 expression. Slope deviations from 0 were considered significant when their *P* value was below 0.05. All correlations were calculated using the Pearson *R*. Significance was then defined as Holm’s corrected *P* value < 0.05.

## Notes

View/request a protocol for this paper from *Bio-protocol*.

## References

[1] Z. Wu, J. M. McGoogan, Characteristics of and important lessons from the coronavirus disease 2019 (COVID-19) outbreak in China. JAMA 323, 1239–1242 (2020). 32091533 10.1001/jama.2020.2648

[2] D. A. Berlin, R. M. Gulick, F. J. Martinez, Severe Covid-19. N. Engl. J. Med. 383, 2451–2460 (2020). 32412710 10.1056/NEJMcp2009575

[3] J. J. Y. Zhang, K. S. Lee, L. W. Ang, Y. S. Leo, B. E. Young, Risk factors for severe disease and efficacy of treatment in patients infected with COVID-19: A systematic review, meta-analysis, and meta-regression analysis. Clin. Infect. Dis. 71, 2199–2206 (2020). 32407459 10.1093/cid/ciaa576 PMC7239203

[4] E. K. Stokes, L. D. Zambrano, K. N. Anderson, E. P. Marder, K. M. Raz, S. el Burai Felix, Y. Tie, K. E. Fullerton, Coronavirus Disease 2019 Case Surveillance—United States, January 22-May 30, 2020. MMWR Morb. Mortal. Wkly Rep. 69, 759–765 (2020). 32555134 10.15585/mmwr.mm6924e2 PMC7302472

[5] J. H. Beigel, K. M. Tomashek, L. E. Dodd, A. K. Mehta, B. S. Zingman, A. C. Kalil, E. Hohmann, H. Y. Chu, A. Luetkemeyer, S. Kline, D. Lopez de Castilla, R. W. Finberg, K. Dierberg, V. Tapson, L. Hsieh, T. F. Patterson, R. Paredes, D. A. Sweeney, W. R. Short, G. Touloumi, D. C. Lye, N. Ohmagari, M. D. Oh, G. M. Ruiz-Palacios, T. Benfield, G. Fätkenheuer, M. G. Kortepeter, R. L. Atmar, C. B. Creech, J. Lundgren, A. G. Babiker, S. Pett, J. D. Neaton, T. H. Burgess, T. Bonnett, M. Green, M. Makowski, A. Osinusi, S. Nayak, H. C. Lane; ACTT-1 Study Group Members, Remdesivir for the treatment of Covid-19—Final report. N. Engl. J. Med. 383, 1813–1826 (2020). 32445440 10.1056/NEJMoa2007764 PMC7262788

[6] H. Gu, Q. Chen, G. Yang, L. He, H. Fan, Y. Q. Deng, Y. Wang, Y. Teng, Z. Zhao, Y. Cui, Y. Li, X. F. Li, J. Li, N. N. Zhang, X. Yang, S. Chen, Y. Guo, G. Zhao, X. Wang, D. Y. Luo, H. Wang, X. Yang, Y. Li, G. Han, Y. He, X. Zhou, S. Geng, X. Sheng, S. Jiang, S. Sun, C. F. Qin, Y. Zhou, Adaptation of SARS-CoV-2 in BALB/c mice for testing vaccine efficacy. Science 369, 1603–1607 (2020). 32732280 10.1126/science.abc4730 PMC7574913

[7] A. Rambaut, N. Loman, O. Pybus, W. Barclay, J. Barrett, A. Carabelli, T. Connor, T. Peacock, D. L. Robertson, E. Volz, on behalf of COVID-19 Genomics Consortium UK. 2020. Preliminary genomic characterization of an emergent SARS-CoV-2 lineage in the UK defined by a novel set of spike mutations.https://virological.org/t/preliminary-genomic-characterisation-of-an-emergent-sars-cov-2-lineage-in-the-uk-defined-by-a-novel-set-of-spike-mutations/563.

[8] C. M. Voloch, R. da Silva Francisco Jr., L. G. P. de Almeida, C. C. Cardoso, O. J. Brustolini, A. L. Gerber, A. P. D. C. Guimarães, D. Mariani, R. M. da Costa, O. C. Ferreira Jr.; Covid19-UFRJ Workgroup; LNCC Workgroup, A. C. Cavalcanti, T. S. Frauches, C. M. B. de Mello, I. de Carvalho Leitão, R. M. Galliez, D. S. Faffe, T. M. P. P. Castiñeiras, A. Tanuri, A. T. R. de Vasconcelos, Genomic characterization of a novel SARS-CoV-2 lineage from Rio de Janeiro, Brazil. J. Virol. 95, e00119-21 (2021). 33649194 10.1128/JVI.00119-21 PMC8139668

[9] WHO, *SARS-CoV-2 Variants* (WHO, 2020).

[10] H. Tegally, E. Wilkinson, M. Giovanetti, A. Iranzadeh, V. Fonseca, J. Giandhari, D. Doolabh, S. Pillay, E. J. San, N. Msomi, K. Mlisana, A. von Gottberg, S. Walaza, M. Allam, A. Ismail, T. Mohale, A. J. Glass, S. Engelbrecht, G. Van Zyl, W. Preiser, F. Petruccione, A. Sigal, D. Hardie, G. Marais, M. Hsiao, S. Korsman, M.-A. Davies, L. Tyers, I. Mudau, D. York, C. Maslo, D. Goedhals, S. Abrahams, O. Laguda-Akingba, A. Alisoltani-Dehkordi, A. Godzik, C. K. Wibmer, B. T. Sewell, J. Lourenço, L. C. J. Alcantara, S. L. K. Pond, S. Weaver, D. Martin, R. J. Lessells, J. N. Bhiman, C. Williamson, T. de Oliveira, Emergence and rapid spread of a new severe acute respiratory syndrome-related coronavirus 2 (SARS-CoV-2) lineage with multiple spike mutations in South Africa. medRxiv 2020.12.21.20248640 (2020).

[11] C. K. Wibmer, F. Ayres, T. Hermanus, M. Madzivhandila, P. Kgagudi, B. Oosthuysen, B. E. Lambson, T. de Oliveira, M. Vermeulen, K. van der Berg, T. Rossouw, M. Boswell, V. Ueckermann, S. Meiring, A. von Gottberg, C. Cohen, L. Morris, J. N. Bhiman, P. L. Moore, SARS-CoV-2 501Y.V2 escapes neutralization by South African COVID-19 donor plasma. Nat. Med. 27, 622–625 (2021). 33654292 10.1038/s41591-021-01285-x

[12] Q. Zhang, P. Bastard, A. Bolze, E. Jouanguy, S.-Y. Zhang; COVID Human Genetic Effort, A. Cobat, L. D. Notarangelo, H. C. Su, L. Abel, J.-L. Casanova, Life-threatening COVID-19: Defective interferons unleash excessive inflammation. Medicine 1, 14–20 (2020). 10.1016/j.medj.2020.12.001 PMC7748410 33363283

[13] Q. Zhang, P. Bastard, Z. Liu, J. L. Pen, M. Moncada-Velez, J. Chen, M. Ogishi, I. K. D. Sabli, S. Hodeib, C. Korol, J. Rosain, K. Bilguvar, J. Ye, A. Bolze, B. Bigio, R. Yang, A. A. Arias, Q. Zhou, Y. Zhang, F. Onodi, S. Korniotis, L. Karpf, Q. Philippot, M. Chbihi, L. Bonnet-Madin, K. Dorgham, N. Smith, W. M. Schneider, B. S. Razooky, H.-H. Hoffmann, E. Michailidis, L. Moens, J. E. Han, L. Lorenzo, L. Bizien, P. Meade, A.-L. Neehus, A. C. Ugurbil, A. Corneau, G. Kerner, P. Zhang, F. Rapaport, Y. Seeleuthner, J. Manry, C. Masson, Y. Schmitt, A. Schlüter, T. L. Voyer, T. Khan, J. Li, J. Fellay, L. Roussel, M. Shahrooei, M. F. Alosaimi, D. Mansouri, H. Al-Saud, F. Al-Mulla, F. Almourfi, S. Z. Al-Muhsen, F. Alsohime, S. A. Turki, R. Hasanato, D. van de Beek, A. Biondi, L. R. Bettini, M. D’Angio, P. Bonfanti, L. Imberti, A. Sottini, S. Paghera, E. Quiros-Roldan, C. Rossi, A. J. Oler, M. F. Tompkins, C. Alba, I. Vandernoot, J.-C. Goffard, G. Smits, I. Migeotte, F. Haerynck, P. Soler-Palacin, A. Martin-Nalda, R. Colobran, P.-E. Morange, S. Keles, F. Çölkesen, T. Ozcelik, K. K. Yasar, S. Senoglu, Ş. N. Karabela, C. Rodríguez-Gallego, G. Novelli, S. Hraiech, Y. Tandjaoui-Lambiotte, X. Duval, C. Laouénan; COVID-STORM Clinicians; COVID Clinicians; Imagine COVID Group; French COVID Cohort Study Group; CoV-Contact Cohort; Amsterdam UMC Covid-19 Biobank; COVID Human Genetic Effort; NIAID-USUHS/TAGC COVID Immunity Group, A. L. Snow, C. L. Dalgard, J. D. Milner, D. C. Vinh, T. H. Mogensen, N. Marr, A. N. Spaan, B. Boisson, S. Boisson-Dupuis, J. Bustamante, A. Puel, M. J. Ciancanelli, I. Meyts, T. Maniatis, V. Soumelis, A. Amara, M. Nussenzweig, A. García-Sastre, F. Krammer, A. Pujol, D. Duffy, R. P. Lifton, S.-Y. Zhang, G. Gorochov, V. Béziat, E. Jouanguy, V. Sancho-Shimizu, C. M. Rice, L. Abel, L. D. Notarangelo, A. Cobat, H. C. Su, J.-L. Casanova, Inborn errors of type I IFN immunity in patients with life-threatening COVID-19. Science 370, eabd4570 (2020). 32972995 10.1126/science.abd4570 PMC7857407

[14] T. Asano, B. Boisson, F. Onodi, D. Matuozzo, M. Moncada-Velez, M. R. L. M. Renkilaraj, P. Zhang, L. Meertens, A. Bolze, M. Materna, S. Korniotis, A. Gervais, E. Talouarn, B. Bigio, Y. Seeleuthner, K. Bilguvar, Y. Zhang, A.-L. Neehus, M. Ogishi, S. J. Pelham, T. L. Voyer, J. Rosain, Q. Philippot, P. Soler-Palacín, R. Colobran, A. Martin-Nalda, J. G. Rivière, Y. Tandjaoui-Lambiotte, K. Chaïbi, M. Shahrooei, I. A. Darazam, N. A. Olyaei, D. Mansouri, N. Hatipoğlu, F. Palabiyik, T. Ozcelik, G. Novelli, A. Novelli, G. Casari, A. Aiuti, P. Carrera, S. Bondesan, F. Barzaghi, P. Rovere-Querini, C. Tresoldi, J. L. Franco, J. Rojas, L. F. Reyes, I. G. Bustos, A. A. Arias, G. Morelle, K. Christèle, J. Troya, L. Planas-Serra, A. Schlüter, M. Gut, A. Pujol, L. M. Allende, C. Rodriguez-Gallego, C. Flores, O. Cabrera-Marante, D. E. Pleguezuelo, Rebeca Pérez de Diego, S. Keles, G. Aytekin, O. M. Akcan, Y. T. Bryceson, P. Bergman, P. Brodin, D. Smole, C I Edvard Smith, A.-C. Norlin, T. M. Campbell, L. E. Covill, L. Hammarström, Q. Pan-Hammarström, H. Abolhassani, S. Mane, N. Marr, M. Ata, F. A. Ali, T. Khan, A. N. Spaan, C. L. Dalgard, P. Bonfanti, A. Biondi, S. Tubiana, C. Burdet, R. Nussbaum, A. Kahn-Kirby, A. L. Snow; COVID Human Genetic Effort; COVID-STORM Clinicians; COVID Clinicians; Imagine COVID Group; French COVID Cohort Study Group; CoV-Contact Cohort; Amsterdam UMC Covid-; Biobank; NIAID-USUHS COVID Study Group, J. Bustamante, A. Puel, S. Boisson-Dupuis, S.-Y. Zhang, V. Béziat, R. P. Lifton, P. Bastard, L. D. Notarangelo, L. Abel, H. C. Su, E. Jouanguy, A. Amara, V. Soumelis, A. Cobat, Q. Zhang, J.-L. Casanova, X-linked recessive TLR7 deficiency in ~1% of men under 60 years old with life-threatening COVID-19. Sci. Immunol. 6, eabl4348 (2021). 34413140 10.1126/sciimmunol.abl4348 PMC8532080

[15] P. Bastard, L. B. Rosen, Q. Zhang, E. Michailidis, H.-H. Hoffmann, Y. Zhang, K. Dorgham, Q. Philippot, J. Rosain, V. Béziat, J. Manry, E. Shaw, L. Haljasmägi, P. Peterson, L. Lorenzo, L. Bizien, S. Trouillet-Assant, K. Dobbs, A. A. de Jesus, A. Belot, A. Kallaste, E. Catherinot, Y. Tandjaoui-Lambiotte, J. L. Pen, G. Kerner, B. Bigio, Y. Seeleuthner, R. Yang, A. Bolze, A. N. Spaan, O. M. Delmonte, M. S. Abers, A. Aiuti, G. Casari, V. Lampasona, L. Piemonti, F. Ciceri, K. Bilguvar, R. P. Lifton, M. Vasse, D. M. Smadja, M. Migaud, J. Hadjadj, B. Terrier, D. Duffy, L. Quintana-Murci, D. van de Beek, L. Roussel, D. C. Vinh, S. G. Tangye, F. Haerynck, D. Dalmau, J. Martinez-Picado, P. Brodin, M. C. Nussenzweig, S. Boisson-Dupuis, C. Rodríguez-Gallego, G. Vogt, T. H. Mogensen, A. J. Oler, J. Gu, P. D. Burbelo, J. I. Cohen, A. Biondi, L. R. Bettini, M. D’Angio, P. Bonfanti, P. Rossignol, J. Mayaux, F. Rieux-Laucat, E. S. Husebye, F. Fusco, M. V. Ursini, L. Imberti, A. Sottini, S. Paghera, E. Quiros-Roldan, C. Rossi, R. Castagnoli, D. Montagna, A. Licari, G. L. Marseglia, X. Duval, J. Ghosn; HGID Lab; NIAID-USUHS Immune Response to COVID Group; COVID Clinicians; COVID-STORM Clinicians; Imagine COVID Group; French COVID Cohort Study Group; Milieu Intérieur Consortium; CoV-Contact Cohort; Amsterdam UMC Covid-19 Biobank; COVID Human Genetic Effort, J. S. Tsang, R. Goldbach-Mansky, K. Kisand, M. S. Lionakis, A. Puel, S.-Y. Zhang, S. M. Holland, G. Gorochov, E. Jouanguy, C. M. Rice, A. Cobat, L. D. Notarangelo, L. Abel, H. C. Su, J.-L. Casanova, Autoantibodies against type I IFNs in patients with life-threatening COVID-19. Science 370, eabd4585 (2020). 32972996 10.1126/science.abd4585 PMC7857397

[16] P. Bastard, A. Gervais, T. Le Voyer, J. Rosain, Q. Philippot, J. Manry, E. Michailidis, H.-H. Hoffmann, S. Eto, M. Garcia-Prat, L. Bizien, A. Parra-Martínez, R. Yang, L. Haljasmägi, M. Migaud, K. Särekannu, J. Maslovskaja, N. de Prost, Y. Tandjaoui-Lambiotte, C.-E. Luyt, B. Amador-Borrero, Alexandre Gaudet, Julien Poissy, P. Morel, P. Richard, F. Cognasse, J. Troya, S. Trouillet-Assant, A. Belot, K. Saker, P. Garçon, J. G. Rivière, J.-C. Lagier, S. Gentile, L. B. Rosen, E. Shaw, T. Morio, J. Tanaka, D. Dalmau, P.-L. Tharaux, D. Sene, A. Stepanian, B. Megarbane, V. Triantafyllia, A. Fekkar, J. R. Heath, J. L. Franco, J.-M. Anaya, J. Solé-Violán, L. Imberti, A. Biondi, P. Bonfanti, R. Castagnoli, O. M. Delmonte, Y. Zhang, A. L. Snow, S. M. Holland, C. Biggs, M. Moncada-Vélez, A. A. Arias, L. Lorenzo, S. Boucherit, B. Coulibaly, D. Anglicheau, A. M. Planas, F. Haerynck, S. Duvlis, R. L. Nussbaum, T. Ozcelik, S. Keles, A. A. Bousfiha, J. E. Bakkouri, C. Ramirez-Santana, S. Paul, Q. Pan-Hammarström, L. Hammarström, A. Dupont, A. Kurolap, C. N. Metz, A. Aiuti, G. Casari, V. Lampasona, F. Ciceri, L. A. Barreiros, E. Dominguez-Garrido, M. Vidigal, M. Zatz, D. van de Beek, S. Sahanic, I. Tancevski, Y. Stepanovskyy, O. Boyarchuk, Y. Nukui, M. Tsumura, L. Vidaur, S. G. Tangye, S. Burrel, D. Duffy, L. Quintana-Murci, A. Klocperk, N. Y. Kann, A. Shcherbina, Y.-L. Lau, D. Leung, M. Coulongeat, J. Marlet, R. Koning, L. F. Reyes, A. Chauvineau-Grenier, F. Venet, G. Monneret, M. C. Nussenzweig, R. Arrestier, I. Boudhabhay, H. Baris-Feldman, D. Hagin, J. Wauters, I. Meyts, A. H. Dyer, S. P. Kennelly, N. M. Bourke, R. Halwani, Narjes Saheb Sharif-Askari, K. Dorgham, J. Sallette, S. M. Sedkaoui, S. A. Khater, R. Rigo-Bonnin, F. Morandeira, L. Roussel, D. C. Vinh, S. R. Ostrowski, A. Condino-Neto, C. Prando, A. Bonradenko, A. N. Spaan, L. Gilardin, J. Fellay, S. Lyonnet, K. Bilguvar, R. P. Lifton, S. Mane; HGID Lab; COVID Clinicians; COVID-STORM Clinicians; NIAID Immune Response to COVID Group; NH-COVAIR Study Group; Danish CHGE; Danish Blood Donor Study; St. James’s Hospital; SARS CoV2 Interest group; French COVID Cohort Study Group; Imagine COVID-Group; Milieu Intérieur Consortium; CoV-Contact Cohort; Amsterdam UMC Covid-19; Biobank Investigators; COVID Human Genetic Effort; CONSTANCES cohort; 3C-Dijon Study; Cerba Health-Care; Etablissement du Sang study group, M. S. Anderson, B. Boisson, V. Béziat, S.-Y. Zhang, E. Vandreakos, O. Hermine, A. Pujol, P. Peterson, T. H. Mogensen, L. Rowen, J. Mond, S. Debette, X. de Lamballerie, X. Duval, F. Mentré, M. Zins, P. Soler-Palacin, R. Colobran, G. Gorochov, X. Solanich, S. Susen, J. Martinez-Picado, D. Raoult, M. Vasse, P. K. Gregersen, L. Piemonti, C. Rodríguez-Gallego, L. D. Notarangelo, H. C. Su, K. Kisand, S. Okada, A. Puel, E. Jouanguy, C. M. Rice, P. Tiberghien, Q. Zhang, A. Cobat, L. Abel, J.-L. Casanova, Autoantibodies neutralizing type I IFNs are present in ~ 4% of uninfected individuals over 70 years old and account for ~ 20% of COVID-19 deaths. Sci. Immunol. 6, abei4340 (2021).

[17] J. Troya, P. Bastard, L. Planas-Serra, P. Ryan, M. Ruiz, M. de Carranza, J. Torres, A. Martínez, L. Abel, J.-L. Casanova, A. Pujol, Neutralizing autoantibodies to type I IFNs in >10% of patients with severe COVID-19 pneumonia hospitalized in Madrid, Spain. J. Clin. Immunol. 41, 914–922 (2021). 33851338 10.1007/s10875-021-01036-0 PMC8043439

[18] S. E. Vazquez, P. Bastard, K. Kelly, A. Gervais, P. J. Norris, L. J. Dumont, J. L. Casanova, M. S. Anderson, J. L. DeRisi, Neutralizing autoantibodies to type I interferons in COVID-19 convalescent donor plasma. J. Clin. Immunol. 41, 1169–1171 (2021). 34009544 10.1007/s10875-021-01060-0 PMC8132742

[19] R. Koning, P. Bastard, J. L. Casanova, M. C. Brouwer, D. van de Beek; with the Amsterdam U.M.C. COVID-19 Biobank Investigators, M. van Agtmael, A. G. Algera, B. Appelman, F. van Baarle, D. Bax, M. Beudel, H. J. Bogaard, M. Bomers, P. Bonta, L. Bos, M. Botta, J. de Brabander, G. Bree, S. de Bruin, M. Bugiani, E. Bulle, N. Chekrouni, O. Chouchane, A. Cloherty, D. A. Dongelmans, R. W. G. Dujardin, P. Elbers, L. Fleuren, S. Geerlings, T. Geijtenbeek, A. Girbes, B. Goorhuis, M. P. Grobusch, F. Hafkamp, L. Hagens, J. Hamann, V. Harris, R. Hemke, S. M. Hermans, L. Heunks, M. Hollmann, J. Horn, J. W. Hovius, M. D. de Jong, R. Koning, E. H. T. Lim, N. van Mourik, J. Nellen, E. J. Nossent, S. Olie, F. Paulus, E. Peters, T. van der Poll, B. Preckel, J. M. Prins, J. Raasveld, T. Reijnders, M. Schinkel, M. J. Schultz, A. Schuurmans, J. Schuurmans, K. Sigaloff, M. A. Slim, M. Smit, C. S. Stijnis, W. Stilma, C. Teunissen, P. Thoral, A. M. Tsonas, M. van der Valk, D. Veelo, H. de Vries, L. A. Vught, M. van Vugt, D. Wouters, A. H. Zwinderman, M. C. Brouwer, W. J. Wiersinga, A. P. J. Vlaar, D. van de Beek, Autoantibodies against type I interferons are associated with multi-organ failure in COVID-19 patients. Intensive Care Med. 47, 704–706 (2021). 33835207 10.1007/s00134-021-06392-4 PMC8034036

[20] G. Beccuti, L. Ghizzoni, V. Cambria, V. Codullo, P. Sacchi, E. Lovati, S. Mongodi, G. A. Iotti, F. Mojoli, A COVID-19 pneumonia case report of autoimmune polyendocrine syndrome type 1 in Lombardy, Italy: Letter to the editor. J. Endocrinol. Investig. 43, 1175–1177 (2020). 32519200 10.1007/s40618-020-01323-4 PMC7282538

[21] A. Meager, K. Visvalingam, P. Peterson, K. Möll, A. Murumägi, K. Krohn, P. Eskelin, J. Perheentupa, E. Husebye, Y. Kadota, N. Willcox, Anti-interferon autoantibodies in autoimmune polyendocrinopathy syndrome type 1. PLOS Med. 3, e289 (2006). 16784312 10.1371/journal.pmed.0030289 PMC1475653

[22] P. Bastard, E. Orlova, L. Sozaeva, R. Lévy, A. James, M. M. Schmitt, S. Ochoa, M. Kareva, Y. Rodina, A. Gervais, T. L. Voyer, J. Rosain, Q. Philippot, A.-L. Neehus, E. Shaw, M. Migaud, L. Bizien, O. Ekwall, S. Berg, G. Beccuti, L. Ghizzoni, G. Thiriez, A. Pavot, C. Goujard, M.-L. Frémond, E. Carter, A. Rothenbuhler, A. Linglart, B. Mignot, A. Comte, N. Cheikh, O. Hermine, L. Breivik, E. S. Husebye, S. Humbert, P. Rohrlich, A. Coaquette, F. Vuoto, K. Faure, N. Mahlaoui, P. Kotnik, T. Battelino, K. T. Podkrajšek, K. Kisand, E. M. N. Ferré, T. D. Maggio, L. B. Rosen, P. D. Burbelo, M. M. Intyre, N. Y. Kann, A. Shcherbina, M. Pavlova, A. Kolodkina, S. M. Holland, S.-Y. Zhang, Y. J. Crow, L. D. Notarangelo, H. C. Su, L. Abel, M. S. Anderson, E. Jouanguy, B. Neven, A. Puel, J.-L. Casanova, M. S. Lionakis, Preexisting autoantibodies to type I IFNs underlie critical COVID-19 pneumonia in patients with APS-1. J. Exp. Med. 218, e20210554 (2021). 33890986 10.1084/jem.20210554 PMC8077172

[23] C. Lucas, P. Wong, J. Klein, T. B. R. Castro, J. Silva, M. Sundaram, M. K. Ellingson, T. Mao, J. E. Oh, B. Israelow, T. Takahashi, M. Tokuyama, P. Lu, A. Venkataraman, A. Park, S. Mohanty, H. Wang, A. L. Wyllie, C. B. F. Vogels, R. Earnest, S. Lapidus, I. M. Ott, A. J. Moore, M. C. Muenker, J. B. Fournier, M. Campbell, C. D. Odio, A. Casanovas-Massana; Yale IMPACT Team, A. Obaid, A. Lu-Culligan, A. Nelson, A. Brito, A. Nunez, A. Martin, A. Watkins, B. Geng, C. Kalinich, C. Harden, C. Todeasa, C. Jensen, D. Kim, D. McDonald, D. Shepard, E. Courchaine, E. B. White, E. Song, E. Silva, E. Kudo, G. DeIuliis, H. Rahming, H. J. Park, I. Matos, J. Nouws, J. Valdez, J. Fauver, J. Lim, K. A. Rose, K. Anastasio, K. Brower, L. Glick, L. Sharma, L. Sewanan, L. Knaggs, M. Minasyan, M. Batsu, M. Petrone, M. Kuang, M. Nakahata, M. Campbell, M. Linehan, M. H. Askenase, M. Simonov, M. Smolgovsky, N. Sonnert, N. Naushad, P. Vijayakumar, R. Martinello, R. Datta, R. Handoko, S. Bermejo, S. Prophet, S. Bickerton, S. Velazquez, T. Alpert, T. Rice, W. Khoury-Hanold, X. Peng, Y. Yang, Y. Cao, Y. Strong, R. Herbst, A. C. Shaw, R. Medzhitov, W. L. Schulz, N. D. Grubaugh, C. dela Cruz, S. Farhadian, A. I. Ko, S. B. Omer, A. Iwasaki, Longitudinal analyses reveal immunological misfiring in severe COVID-19. Nature 584, 463–469 (2020). 32717743 10.1038/s41586-020-2588-y PMC7477538

[24] J. P. Bernardes, N. Mishra, F. Tran, T. Bahmer, L. Best, J. I. Blase, D. Bordoni, J. Franzenburg, U. Geisen, J. Josephs-Spaulding, P. Köhler, A. Künstner, E. Rosati, A. C. Aschenbrenner, P. Bacher, N. Baran, T. Boysen, B. Brandt, N. Bruse, J. Dörr, A. Dräger, G. Elke, D. Ellinghaus, J. Fischer, M. Forster, A. Franke, S. Franzenburg, N. Frey, A. Friedrichs, J. Fuß, A. Glück, J. Hamm, F. Hinrichsen, M. P. Hoeppner, S. Imm, R. Junker, S. Kaiser, Y. H. Kan, R. Knoll, C. Lange, G. Laue, C. Lier, M. Lindner, G. Marinos, R. Markewitz, J. Nattermann, R. Noth, P. Pickkers, K. F. Rabe, A. Renz, C. Röcken, J. Rupp, A. Schaffarzyk, A. Scheffold, J. Schulte-Schrepping, D. Schunk, D. Skowasch, T. Ulas, K.-P. Wandinger, M. Wittig, J. Zimmermann, H. Busch, B. F. Hoyer, C. Kaleta, J. Heyckendorf, M. Kox, J. Rybniker, S. Schreiber, J. L. Schultze, P. Rosenstiel; HCA Lung Biological Network; Deutsche COVID-19 Omics Initiative (DeCOI), Longitudinal multi-omics analyses identify responses of megakaryocytes, erythroid cells, and plasmablasts as hallmarks of severe COVID-19. Immunity 53, 1296–1314.e9 (2020). 33296687 10.1016/j.immuni.2020.11.017 PMC7689306

[25] A. J. Combes, T. Courau, N. F. Kuhn, K. H. Hu, A. Ray, W. S. Chen, N. W. Chew, S. J. Cleary, D. Kushnoor, G. C. Reeder, A. Shen, J. Tsui, K. J. Hiam-Galvez, P. Muñoz-Sandoval, W. S. Zhu, D. S. Lee, Y. Sun, R. You, M. Magnen, L. Rodriguez, K. W. Im, N. K. Serwas, A. Leligdowicz, C. R. Zamecnik, R. P. Loudermilk, M. R. Wilson, C. J. Ye, G. K. Fragiadakis, M. R. Looney, V. Chan, A. Ward, S. Carrillo; The UCSF COMET Consortium, C. Cathy, J. Zhan, B. Samad, S. Chak, R. Ghale, J. Giberson, A. Gonzalez, A. Jauregui, D. Lee, V. Nguyen, K. Yee, Y. Abe-Jones, L. Pierce, P. Prasad, P. Sinha, A. Beagle, T. Lea, A. Esmalii, A. Sigman, G. M. Ortiz, K. Raffel, C. Jones, K. Liu, W. Eckalbar, M. Matthay, D. J. Erle, P. G. Woodruff, C. Langelier, K. Kangelaris, C. M. Hendrickson, C. Calfee, A. A. Rao, M. F. Krummel, Global absence and targeting of protective immune states in severe COVID-19. Nature 591, 124–130 (2021). 33494096 10.1038/s41586-021-03234-7 PMC8567458

[26] NIH, *Clinical Spectrum of SARS-CoV-2 Infection* (NIH, 2020).

[27] G. Chamie, C. Marquez, E. Crawford, J. Peng, M. Petersen, D. Schwab, J. Schwab, J. Martinez, D. Jones, D. Black, M. Gandhi, A. D. Kerkhoff, V. Jain, F. Sergi, J. Jacobo, S. Rojas, V. Tulier-Laiwa, T. Gallardo-Brown, A. Appa, C. Chiu, M. Rodgers, J. Hackett Jr.; CLIAhub Consortium, A. Kistler, S. Hao, J. Kamm, D. Dynerman, J. Batson, B. Greenhouse, J. De Risi, D. V. Havlir, SARS-CoV-2 community transmission disproportionately affects Latinx population during shelter-in-place in San Francisco. Clin. Infect. Dis. 2020, ciaa1234 (2020). 10.1093/cid/ciaa1234 PMC7499499 32821935

[28] A. J. Wilk, A. Rustagi, N. Q. Zhao, J. Roque, G. J. Martínez-Colón, J. L. McKechnie, G. T. Ivison, T. Ranganath, R. Vergara, T. Hollis, L. J. Simpson, P. Grant, A. Subramanian, A. J. Rogers, C. A. Blish, A single-cell atlas of the peripheral immune response in patients with severe COVID-19. Nat. Med. 26, 1070–1076 (2020). 32514174 10.1038/s41591-020-0944-y PMC7382903

[29] E. Stephenson, G. Reynolds, R. A. Botting, F. J. Calero-Nieto, M. D. Morgan, Z. K. Tuong, K. Bach, W. Sungnak, K. B. Worlock, M. Yoshida, N. Kumasaka, K. Kania, J. Engelbert, B. Olabi, J. S. Spegarova, N. K. Wilson, N. Mende, L. Jardine, L. C. S. Gardner, I. Goh, D. Horsfall, J. M. Grath, S. Webb, M. W. Mather, R. G. H. Lindeboom, E. Dann, N. Huang, K. Polanski, E. Prigmore, F. Gothe, J. Scott, R. P. Payne, K. F. Baker, A. T. Hanrath, I. C. D. S. van der Loeff, A. S. Barr, A. Sanchez-Gonzalez, L. Bergamaschi, F. Mescia, J. L. Barnes, E. Kilich, A. de Wilton, A. Saigal, A. Saleh, S. M. Janes, C. M. Smith, N. Gopee, C. Wilson, P. Coupland, J. M. Coxhead, V. Y. Kiselev, S. van Dongen, J. Bacardit, H. W. King; Cambridge Institute of Therapeutic Immunology, Infectious Disease-National Institute of Health Research (CITIID-NIHR) COVID-19 Bio Resource Collaboration, A. J. Rostron, A. J. Simpson, S. Hambleton, E. Laurenti, P. A. Lyons, K. B. Meyer, M. Z. Nikolić, C. J. A. Duncan, K. G. C. Smith, S. A. Teichmann, M. R. Clatworthy, J. C. Marioni, B. Göttgens, M. Haniffa, Single-cell multi-omics analysis of the immune response in COVID-19. Nat. Med. 27, 904–916 (2021). 33879890 10.1038/s41591-021-01329-2 PMC8121667

[30] C. Liu, A. J. Martins, W. W. Lau, N. Rachmaninoff, J. Chen, L. Imberti, D. Mostaghimi, D. L. Fink, P. D. Burbelo, K. Dobbs, O. M. Delmonte, N. Bansal, L. Failla, A. Sottini, E. Quiros-Roldan, K. L. Han, B. A. Sellers, F. Cheung, R. Sparks, T.-W. Chun, S. Moir, M. S. Lionakis; NIAID COVID Consortium; COVID Clinicians, C. Rossi, H. C. Su, D. B. Kuhns, J. I. Cohen, L. D. Notarangelo, J. S. Tsang, Time-resolved systems immunology reveals a late juncture linked to fatal COVID-19. Cell 184, 1836–1857.e22 (2021). 33713619 10.1016/j.cell.2021.02.018 PMC7874909

[31] M. O. Ainle, L. Helms, J. Vermeire, F. Roesch, D. Humes, R. Basom, J. J. Delrow, J. Overbaugh, M. Emerman, A virus-packageable CRISPR screen identifies host factors mediating interferon inhibition of HIV. eLife 7, e39823 (2018). 30520725 10.7554/eLife.39823 PMC6286125

[32] T. H. C. Brondijk, T. de Ruiter, J. Ballering, H. Wienk, R. J. Lebbink, H. van Ingen, R. Boelens, R. W. Farndale, L. Meyaard, E. G. Huizinga, Crystal structure and collagen-binding site of immune inhibitory receptor LAIR-1: Unexpected implications for collagen binding by platelet receptor GPVI. Blood 115, 1364–1373 (2010). 20007810 10.1182/blood-2009-10-246322

[33] R. J. Lebbink, T. de Ruiter, J. Adelmeijer, A. B. Brenkman, J. M. van Helvoort, M. Koch, R. W. Farndale, T. Lisman, A. Sonnenberg, P. J. Lenting, L. Meyaard, Collagens are functional, high affinity ligands for the inhibitory immune receptor LAIR-1. J. Exp. Med. 203, 1419–1425 (2006). 16754721 10.1084/jem.20052554 PMC2118306

[34] L. Meyaard, J. Hurenkamp, H. Clevers, L. L. Lanier, J. H. Phillips, Leukocyte-associated Ig-like receptor-1 functions as an inhibitory receptor on cytotoxic T cells. J. Immunol. 162, 5800–5804 (1999). 10229813

[35] R. E. Lanford, B. Guerra, H. Lee, D. Chavez, K. M. Brasky, C. B. Bigger, Genomic response to interferon-α in chimpanzees: Implications of rapid downregulation for hepatitis C kinetics. Hepatology 43, 961–972 (2006). 16628626 10.1002/hep.21167

[36] L. Ren, L. Zhang, D. Chang, J. Wang, Y. Hu, H. Chen, L. Guo, C. Wu, C. Wang, Y. Wang, Y. Wang, G. Wang, S. Yang, C. S. dela Cruz, L. Sharma, L. Wang, D. Zhang, J. Wang, The kinetics of humoral response and its relationship with the disease severity in COVID-19. Commun. Biol. 3, 780 (2020). 33311543 10.1038/s42003-020-01526-8 PMC7733479

[37] G. Xu, F. Qi, H. Li, Q. Yang, H. Wang, X. Wang, X. Liu, J. Zhao, X. Liao, Y. Liu, L. Liu, S. Zhang, Z. Zhang, The differential immune responses to COVID-19 in peripheral and lung revealed by single-cell RNA sequencing. Cell Discov. 6, 73 (2020). 33101705 10.1038/s41421-020-00225-2 PMC7574992

[38] J. Schulte-Schrepping, N. Reusch, D. Paclik, K. Baßler, S. Schlickeiser, B. Zhang, B. Krämer, T. Krammer, S. Brumhard, L. Bonaguro, E. De Domenico, D. Wendisch, M. Grasshoff, T. S. Kapellos, M. Beckstette, T. Pecht, A. Saglam, O. Dietrich, H. E. Mei, A. R. Schulz, C. Conrad, D. Kunkel, E. Vafadarnejad, C.-J. Xu, A. Horne, M. Herbert, A. Drews, C. Thibeault, M. Pfeiffer, S. Hippenstiel, A. Hocke, H. Müller-Redetzky, K.-M. Heim, F. Machleidt, A. Uhrig, L. B. de Jarcy, L. Jürgens, M. Stegemann, C. R. Glösenkamp, H.-D. Volk, C. Goffinet, M. Landthaler, E. Wyler, P. Georg, M. Schneider, C. Dang-Heine, N. Neuwinger, K. Kappert, R. Tauber, V. Corman, J. Raabe, K. M. Kaiser, M. T. Vinh, G. Rieke, C. Meisel, T. Ulas, M. Becker, R. Geffers, M. Witzenrath, C. Drosten, N. Suttorp, C. von Kalle, F. Kurth, K. Händler, J. L. Schultze, A. C. Aschenbrenner, Y. Li, J. Nattermann, B. Sawitzki, A.-E. Saliba, L. E. Sander; Deutsche COVID-19 OMICS Initiative (DeCOI), Severe COVID-19 is marked by a dysregulated myeloid cell compartment. Cell 182, 1419–1440.e23 (2020). 32810438 10.1016/j.cell.2020.08.001 PMC7405822

[39] E. Y. Wang, T. Mao, J. Klein, Y. Dai, J. D. Huck, J. R. Jaycox, F. Liu, T. Zhou, B. Israelow, P. Wong, A. Coppi, C. Lucas, J. Silva, J. E. Oh, E. Song, E. S. Perotti, N. S. Zheng, S. Fischer, M. Campbell, J. B. Fournier, A. L. Wyllie, C. B. F. Vogels, I. M. Ott, C. C. Kalinich, M. E. Petrone, A. E. Watkins; Yale IMPACT Team, A. Obaid, A. J. Moore, A. Casanovas-Massana, A. Lu-Culligan, A. Nelson, A. Nunez, A. Martin, B. Geng, C. D. Odio, C. A. Harden, C. Todeasa, C. Jensen, D. Kim, D. McDonald, D. Shepard, E. Courchaine, E. B. White, E. Silva, E. Kudo, G. DeIuliis, H. Rahming, H. J. Park, I. Matos, J. Nouws, J. Valdez, J. Lim, K. A. Rose, K. Anastasio, K. Brower, L. Glick, L. Sharma, L. Sewanan, L. Knaggs, M. Minasyan, M. Batsu, M. Kuang, M. Nakahata, M. Linehan, M. H. Askenase, M. Simonov, M. Smolgovsky, N. Sonnert, N. Naushad, P. Vijayakumar, R. Martinello, R. Datta, R. Handoko, S. Bermejo, S. Prophet, S. Bickerton, S. Velazquez, T. Rice, W. Khoury-Hanold, X. Peng, Y. Yang, Y. Cao, Y. Strong, C. dela Cruz, S. F. Farhadian, W. L. Schulz, S. Ma, N. D. Grubaugh, A. I. Ko, A. Iwasaki, A. M. Ring, Diverse functional autoantibodies in patients with COVID-19. Nature 595, 283–288 (2021). 34010947 10.1038/s41586-021-03631-y PMC13130511

[40] A. Poggi, N. Pella, L. Morelli, F. Spada, V. Revello, S. Sivori, R. Augugliaro, L. Moretta, A. Moretta, P40, a novel surface molecule involved in the regulation of the non-major histocompatibility complex-restricted cytolytic activity in humans. Eur. J. Immunol. 25, 369–376 (1995). 7875198 10.1002/eji.1830250210

[41] L. Meyaard, G. J. Adema, C. Chang, E. Woollatt, G. R. Sutherland, L. L. Lanier, J. H. Phillips, LAIR-1, a novel inhibitory receptor expressed on human mononuclear leukocytes. Immunity 7, 283–290 (1997). 9285412 10.1016/s1074-7613(00)80530-0

[42] I. Bonaccorsi, C. Cantoni, P. Carrega, D. Oliveri, G. Lui, R. Conte, M. Navarra, R. Cavaliere, E. Traggiai, M. Gattorno, A. Martini, M. C. Mingari, A. Moretta, G. Ferlazzo, The immune inhibitory receptor lair-1 is highly expressed by plasmacytoid dendritic cells and acts complementary with NKp44 to control IFNα production. PLOS ONE 5, e15080 (2010). 21151495 10.1371/journal.pone.0015080 PMC2994815

[43] M. Son, B. Diamond, C1q-mediated repression of human monocytes is regulated by leukocyte-associated Ig-like receptor 1 (LAIR-1). Mol. Med. 20, 559–568 (2014). 10.2119/molmed.2014.00185 PMC4365071 25247291

[44] E. M. N. Ferre, S. R. Rose, S. D. Rosenzweig, P. D. Burbelo, K. R. Romito, J. E. Niemela, L. B. Rosen, T. J. Break, W. Gu, S. Hunsberger, S. K. Browne, A. P. Hsu, S. Rampertaap, M. Swamydas, A. L. Collar, H. H. Kong, C.-C. R. Lee, D. Chascsa, T. Simcox, A. Pham, A. Bondici, M. Natarajan, J. Monsale, D. E. Kleiner, M. Quezado, I. Alevizos, N. M. Moutsopoulos, L. Yockey, C. Frein, A. Soldatos, K. R. Calvo, J. Adjemian, M. N. Similuk, D. M. Lang, K. D. Stone, G. Uzel, J. B. Kopp, R. J. Bishop, S. M. Holland, K. N. Olivier, T. A. Fleisher, T. Heller, K. K. Winer, M. S. Lionakis, Redefined clinical features and diagnostic criteria in autoimmune polyendocrinopathy-candidiasis-ectodermal dystrophy. JCI Insight 1, e88782 (2016). 27588307 10.1172/jci.insight.88782 PMC5004733

[45] E. M. N. Ferré, T. J. Break, P. D. Burbelo, M. Allgäuer, D. E. Kleiner, D. Jin, Z. Xu, L. R. Folio, D. J. Mollura, M. Swamydas, W. Gu, S. Hunsberger, C.-C. R. Lee, A. Bondici, K. W. Hoffman, J. K. Lim, K. Dobbs, J. E. Niemela, T. A. Fleisher, A. P. Hsu, L. N. Snow, D. N. Darnell, S. Ojaimi, M. A. Cooper, M. Bozzola, G. I. Kleiner, J. C. Martinez, R. R. Deterding, D. B. Kuhns, T. Heller, K. K. Winer, A. Rajan, S. M. Holland, L. D. Notarangelo, K. P. Fennelly, K. N. Olivier, M. S. Lionakis, Lymphocyte-driven regional immunopathology in pneumonitis caused by impaired central immune tolerance. Sci. Transl. Med. 11, eaav5597 (2019). 31167928 10.1126/scitranslmed.aav5597 PMC6647037

[46] H. M. Kang, M. Subramaniam, S. Targ, M. Nguyen, L. Maliskova, E. McCarthy, E. Wan, S. Wong, L. Byrnes, C. M. Lanata, R. E. Gate, S. Mostafavi, A. Marson, N. Zaitlen, L. A. Criswell, C. J. Ye, Multiplexed droplet single-cell RNA-sequencing using natural genetic variation. Nat. Biotechnol. 36, 89–94 (2018). 29227470 10.1038/nbt.4042 PMC5784859

[47] G. X. Y. Zheng, J. M. Terry, P. Belgrader, P. Ryvkin, Z. W. Bent, R. Wilson, S. B. Ziraldo, T. D. Wheeler, G. P. McDermott, J. Zhu, M. T. Gregory, J. Shuga, L. Montesclaros, J. G. Underwood, D. A. Masquelier, S. Y. Nishimura, M. Schnall-Levin, P. W. Wyatt, C. M. Hindson, R. Bharadwaj, A. Wong, K. D. Ness, L. W. Beppu, H. J. Deeg, C. McFarland, K. R. Loeb, W. J. Valente, N. G. Ericson, E. A. Stevens, J. P. Radich, T. S. Mikkelsen, B. J. Hindson, J. H. Bielas, Massively parallel digital transcriptional profiling of single cells. Nat. Commun. 8, 14049 (2017). 28091601 10.1038/ncomms14049 PMC5241818

[48] The 1000 Genomes Project Consortium, A global reference for human genetic variation. Nature 526, 68–74 (2015). 26432245 10.1038/nature15393 PMC4750478

[49] F. A. Wolf, P. Angerer, F. J. Theis, SCANPY: Large-scale single-cell gene expression data analysis. Genome Biol. 19, 15 (2018). 29409532 10.1186/s13059-017-1382-0 PMC5802054

[50] W. E. Johnson, C. Li, A. Rabinovic, Adjusting batch effects in microarray expression data using empirical Bayes methods. Biostatistics 8, 118–127 (2006). 16632515 10.1093/biostatistics/kxj037

[51] D. V. Klopfenstein, L. Zhang, B. S. Pedersen, F. Ramírez, A. Warwick Vesztrocy, A. Naldi, C. J. Mungall, J. M. Yunes, O. Botvinnik, M. Weigel, W. Dampier, C. Dessimoz, P. Flick, H. Tang, GOATOOLS: A Python library for Gene Ontology analyses. Sci. Rep. 8, 10872 (2018). 30022098 10.1038/s41598-018-28948-z PMC6052049

[52] E. D. Crawford, I. Acosta, V. Ahyong, E. C. Anderson, S. Arevalo, D. Asarnow, S. Axelrod, P. Ayscue, C. S. Azimi, C. M. Azumaya, S. Bachl, I. Bachmutsky, A. Bhaduri, J. B. Brown, J. Batson, A. Behnert, R. M. Boileau, S. R. Bollam, A. R. Bonny, D. Booth, M. J. B. Borja, D. Brown, B. Buie, C. E. Burnett, L. E. Byrnes, K. A. Cabral, J. P. Cabrera, S. Caldera, G. Canales, G. R. Castañeda, A. P. Chan, C. R. Chang, A. Charles-Orszag, C. Cheung, U. Chio, E. D. Chow, Y. R. Citron, A. Cohen, L. B. Cohn, C. Chiu, M. A. Cole, D. N. Conrad, A. Constantino, A. Cote, T.’. J. Crayton-Hall, S. Darmanis, A. M. Detweiler, R. L. Dial, S. Dong, E. M. Duarte, D. Dynerman, R. Egger, A. Fanton, S. M. Frumm, B. X. H. Fu, V. E. Garcia, J. Garcia, C. Gladkova, M. Goldman, R. Gomez-Sjoberg, M. G. Gordon, J. C. R. Grove, S. Gupta, A. Haddjeri-Hopkins, P. Hadley, J. Haliburton, S. L. Hao, G. Hartoularos, N. Herrera, M. Hilberg, K. Y. E. Ho, N. Hoppe, S. Hosseinzadeh, C. J. Howard, J. A. Hussmann, E. Hwang, D. Ingebrigtsen, J. R. Jackson, Z. M. Jowhar, D. Kain, J. Y. S. Kim, A. Kistler, O. Kreutzfeld, J. Kulsuptrakul, A. F. Kung, C. Langelier, M. T. Laurie, L. Lee, K. Leng, K. E. Leon, M. D. Leonetti, S. R. Levan, S. Li, A. W. Li, J. Liu, H. S. Lubin, A. Lyden, J. Mann, S. Mann, G. Margulis, D. M. Marquez, B. P. Marsh, C. Martyn, E. E. McCarthy, A. McGeever, A. F. Merriman, L. K. Meyer, S. Miller, M. K. Moore, C. T. Mowery, T. Mukhtar, L. L. Mwakibete, N. Narez, N. F. Neff, L. A. Osso, D. Oviedo, S. Peng, M. Phelps, K. Phong, P. Picard, L. M. Pieper, N. Pincha, A. O. Pisco, A. Pogson, S. Pourmal, R. R. Puccinelli, A. S. Puschnik, E. Rackaityte, P. Raghavan, M. Raghavan, J. Reese, J. M. Replogle, H. Retallack, H. Reyes, D. Rose, M. F. Rosenberg, E. Sanchez-Guerrero, S. M. Sattler, L. Savy, S. K. See, K. K. Sellers, P. H. Serpa, M. Sheehy, J. Sheu, S. Silas, J. A. Streithorst, J. Strickland, D. Stryke, S. Sunshine, P. Suslow, R. Sutanto, S. Tamura, M. Tan, J. Tan, A. Tang, C. M. Tato, J. C. Taylor, I. Tenvooren, E. M. Thompson, E. C. Thornborrow, E. Tse, T. Tung, M. L. Turner, V. S. Turner, R. E. Turnham, M. J. Turocy, T. V. Vaidyanathan, I. D. Vainchtein, M. Vanaerschot, S. E. Vazquez, A. M. Wandler, A. Wapniarski, J. T. Webber, Z. Y. Weinberg, A. Westbrook, A. W. Wong, E. Wong, G. Worthington, F. Xie, A. Xu, T. Yamamoto, Y. Yang, F. Yarza, Y. Zaltsman, T. Zheng, J. L. DeRisi, Rapid deployment of SARS-CoV-2 testing: The CLIAHUB. PLOS Pathog. 16, e1008966 (2020). 33112933 10.1371/journal.ppat.1008966 PMC7592773

[53] N. Farbehi, R. Patrick, A. Dorison, M. Xaymardan, V. Janbandhu, K. Wystub-Lis, J. W. K. Ho, R. E. Nordon, R. P. Harvey, Single-cell expression profiling reveals dynamic flux of cardiac stromal, vascular and immune cells in health and injury. eLife 8, e43882 (2019). 30912746 10.7554/eLife.43882 PMC6459677

