# Supplementary material for: Type I interferon autoantibodies are associated with systemic immune alterations in patients with COVID-19
Source: Sci Transl Med. 2021 Aug 24;13(612):eabh2624. doi: 10.1126/scitranslmed.abh2624 (PMC8601717; doi:10.1126/scitranslmed.abh2624)
Supplement: Supplementary file 1 — Figs. S1 to S6 [file scitranslmed.abh2624_sm.pdf]

Science Translational Medicine
AAAS

Supplementary Materials for

Type I interferon autoantibodies are associated with systemic immune alterations in patients with COVID-19

Monique G. P. van der Wijst *et al.*

Corresponding author. Email: Jean-Laurent Casanova, casanova@rockefeller.edu; Joseph L. Derisi, joe@derisilab.ucsf.edu; Mark S. Anderson, mark.anderson@ucsf.edu; Chun Jimmie Ye, jimmie.ye@ucsf.edu

Sci. Transl. Med. **13**, eabh2624 (2021)
DOI: 10.1126/scitranslmed.abh2624

The PDF file includes:

Figs. S1 to S6

Other Supplementary Material for this manuscript includes the following:

Data files S1 to S9

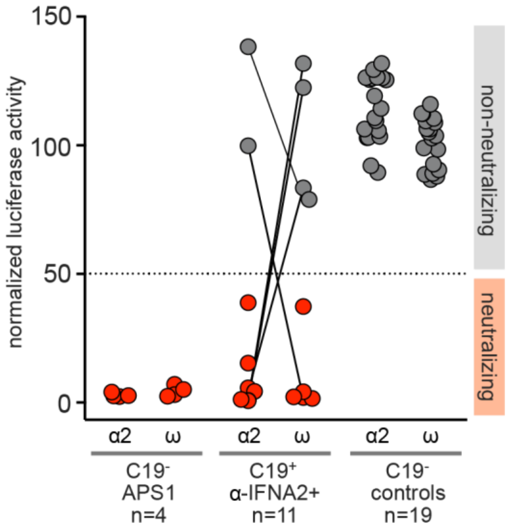

Figure S1. Anti-IFN antibodies have neutralizing capacity. Neutralization of IFN-α2 and IFN-ω in the presence of 10% plasma of Autoimmune Polyglandular Syndrome Type 1 (APS-1) patients (left), patients with severe-to-critical Coronavirus Disease 2019 (COVID-19) and auto-Abs against IFN-α2 as identified by radiolabeled antigens (RLBA) (middle) or healthy uninfected controls (right). For donors where either IFN-α2 or IFN-ω neutralization was negative, paired samples are connected by a line, highlighting that only a single sample was negative for neutralization against both cytokine subsets (corresponding to the single RLBA anti-IFN-α2 positive sample without a neutralizing arrow in **Fig. 1A**). Relative luciferase activity is shown (ISRE dual luciferase activity normalized to Renilla) after stimulation with 10 ng/ml of IFN-α2 or IFN-ω. ISRE, IFN stimulation response element.

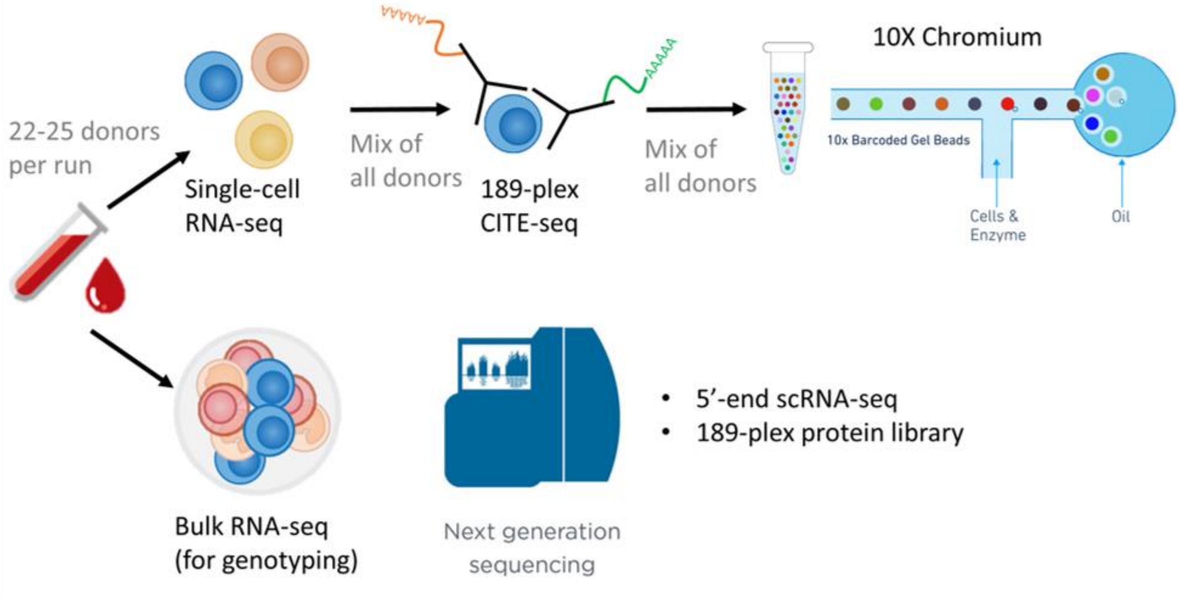

Figure S2. Multiplexed single-cell epitope and transcriptome sequencing was performed in patients with and without COVID-19 and COVID-19, and healthy controls. Across 9 runs of 22 to 25 donors, frozen peripheral blood mononuclear cells (PBMCs) were thawed, multiplexed, stained, and processed using cellular indexing of transcriptomes and epitopes by sequencing (CITE-seq).

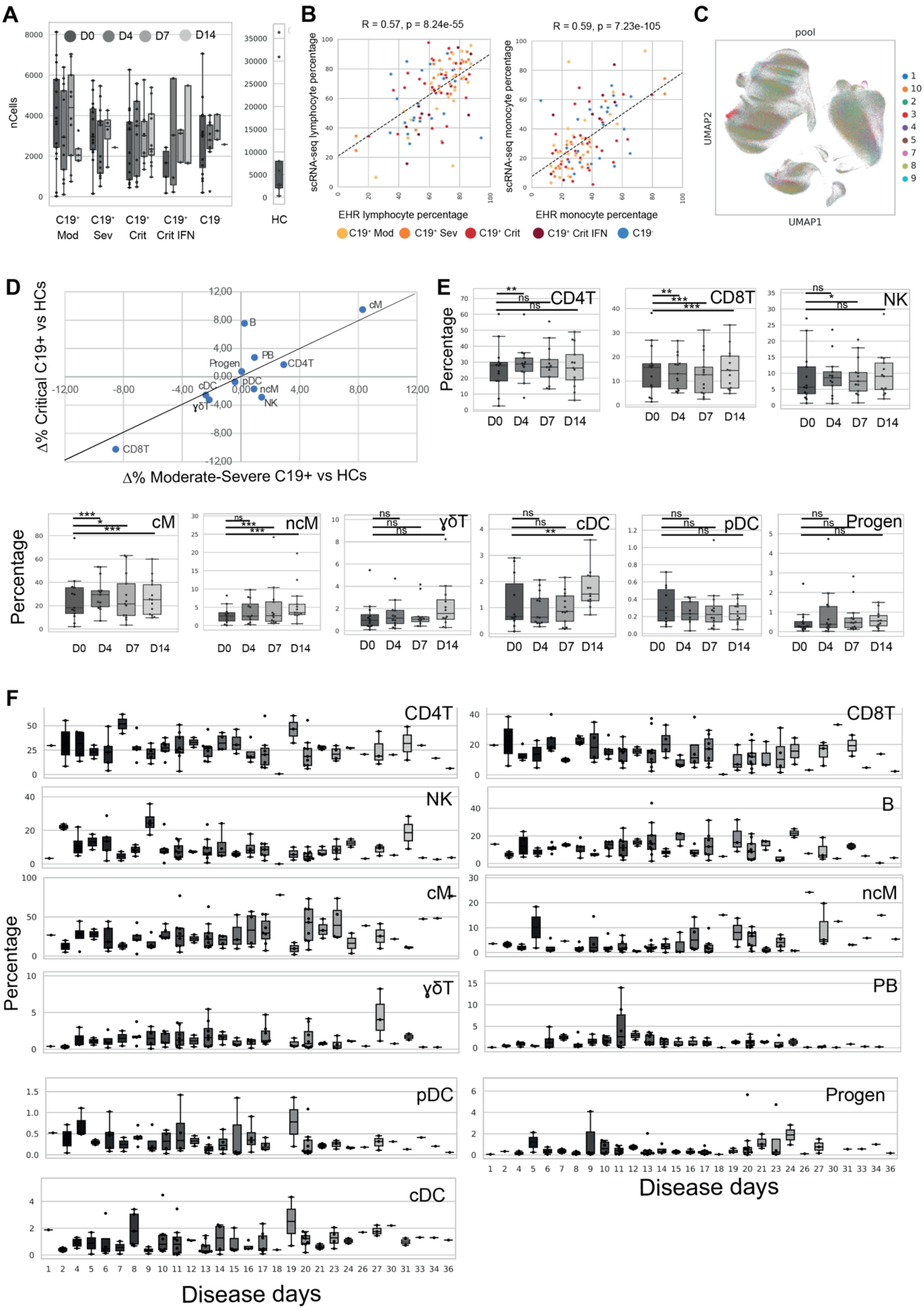

A
D0 D4 D7 D14
nCells
C19+ Mod
C19+ Sev
C19+ Crit
C19+ Crit IFN
C19-
HC
B
R = 0.57, p = 8.24e-55
scRNA-seq lymphocyte percentage
EHR lymphocyte percentage
R = 0.59, p = 7.23e-105
scRNA-seq monocyte percentage
EHR monocyte percentage
C19+ Mod
C19+ Sev
C19+ Crit
C19+ Crit IFN
C19-
C
pool
UMAP2
UMAP1
1 10 2 3 4 5 7 8 9
D
Δ% Critical C19+ vs HCs
Δ% Moderate-Severe C19+ vs HCs
cM
B
PB
Progen
CD4T
pDC
cDC
ncM
NK
γδT
CD8T
E
Percentage
CD4T
CD8T
NK
cM
ncM
γδT
cDC
pDC
Progen
D0 D4 D7 D14
F
CD4T
CD8T
NK
B
cM
ncM
γδT
PB
pDC
Progen
cDC
Percentage
Disease days

Figure S3. Shifts in circulating leukocyte composition are observed in samples isolated from individuals with critical COVID-19. (A) Average number of cells remaining after quality control (QC) is shown per donor per timepoint per disease severity status is shown. **(B)** Pearson correlation between electronic health record (EHR) and scRNA-seq derived blood counts for lymphocytes (left) and monocytes (right). Each datapoint is colored by disease severity status. **(C)** Uniform Manifold Approximation and Projection (UMAP) of PBMCs colored by experimental run. **(D)** Scatterplot showing the absolute percentage composition difference of moderate-severe (x-axis) or critical COVID-19^{+} cases (y-axis) versus healthy controls. Each datapoint shows the donor average per cell type. The dotted line depicts the situation in which a cell type would have similar changes in both moderate-severe and critical COVID-19^{+} cases as opposed to healthy controls. **(E)** Boxplots of the cell type percentages (y-axis) in patients with COVID-19 over day 0, 4, 7 and 14 since hospitalization (D0, D4, D7, D14) or **(F)** over day since first symptoms. Significant differences are presented using Holm's multiple-testing corrected, permutation-based p-values: *** $p < 0.001$, ** $p < 0.01$, * $p < 0.05$, ns = not significant.

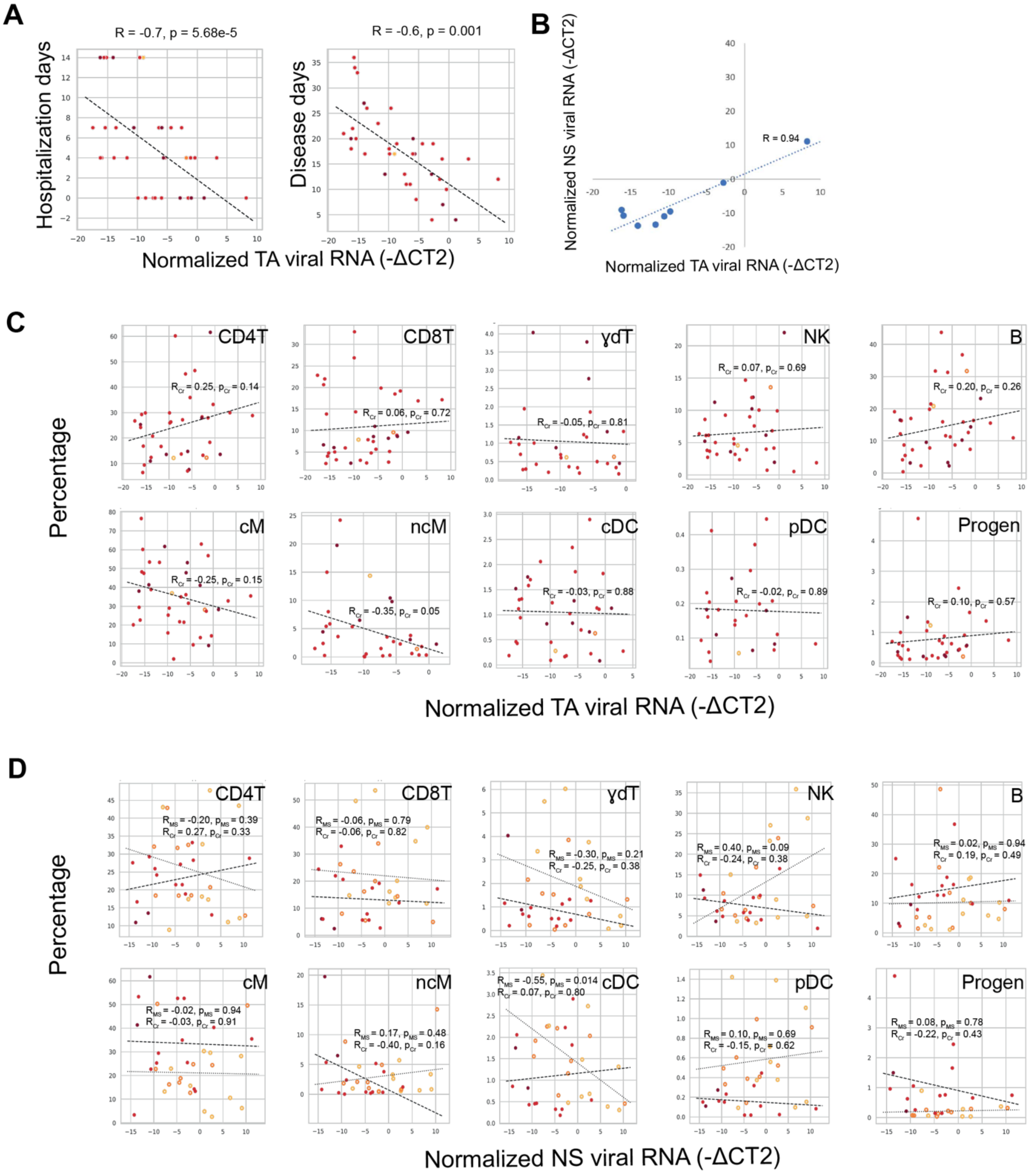

Figure S4. Circulating leukocyte composition correlates with SARS-CoV-2 titers in COVID-19. (A) Scatterplot of SARS-CoV-2 viral RNA abundance as measured by real-time quantitative reverse transcription polymerase chain reaction (qRT-PCR) in tracheal aspirates (inverse ΔCT, x-axis) and days since hospitalization (left) or days since first symptoms (rights) (R = Pearson correlation). **(B)** Correlation between viral RNA abundance as measured by qRT-PCR in tracheal aspirates (x-axis) and nasal swabs (y-axis), as measured in those samples for which both sample types were available. **(C and D)** Scatterplots depict SARS-CoV-2 viral RNA abundance as measured by qRT-PCR in tracheal aspirates (C) or nasal swabs (D) and cell type compositions. Pearson correlations are shown as measured in all critical (R_{Cr}) or moderate-severe cases (R_{MS}).

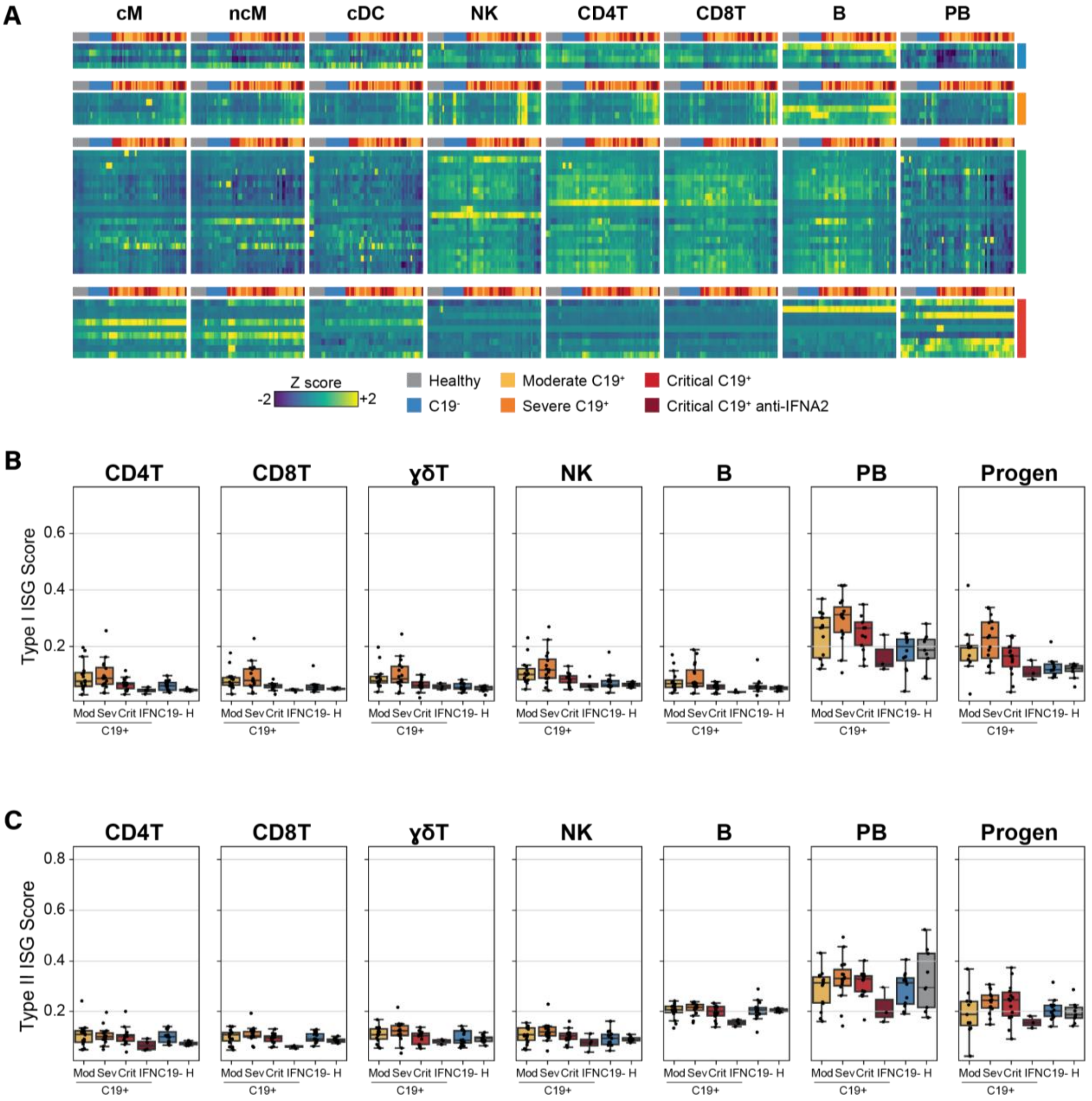

Figure S5. Surface protein expression and ISG response change in leukocyte subsets in critical COVID-19. (A) Heatmaps show 38 surface proteins differentially expressed at day 0 (FDR < 0.05, |log(fold change)| >0) in at least one of the 11 cell types. CD4$^+$ T cells (CD4T), CD8$^+$ T cells (CD8T), natural killer cells (NK), B cells (B), plasmablasts (PB), classical monocytes (cM), non-classical monocytes (ncM), and conventional dendritic cells (cDC) are shown. Each row represents a surface protein and each column is the average expression of the proteins in a particular sample across all cells of a specific type. Samples are grouped by case-control status and COVID-19 severity. Expression is row standardized. **(B)** Type I-specific ISG score (y-axis) at day 0 across 8 cell types separated by case-control status and disease severity. **(C)** Type II-specific ISG score (y-axis) at day 0 across 8 cell types separated by case-control status and disease severity. Boxplots show median, 25th and 75th percentiles.

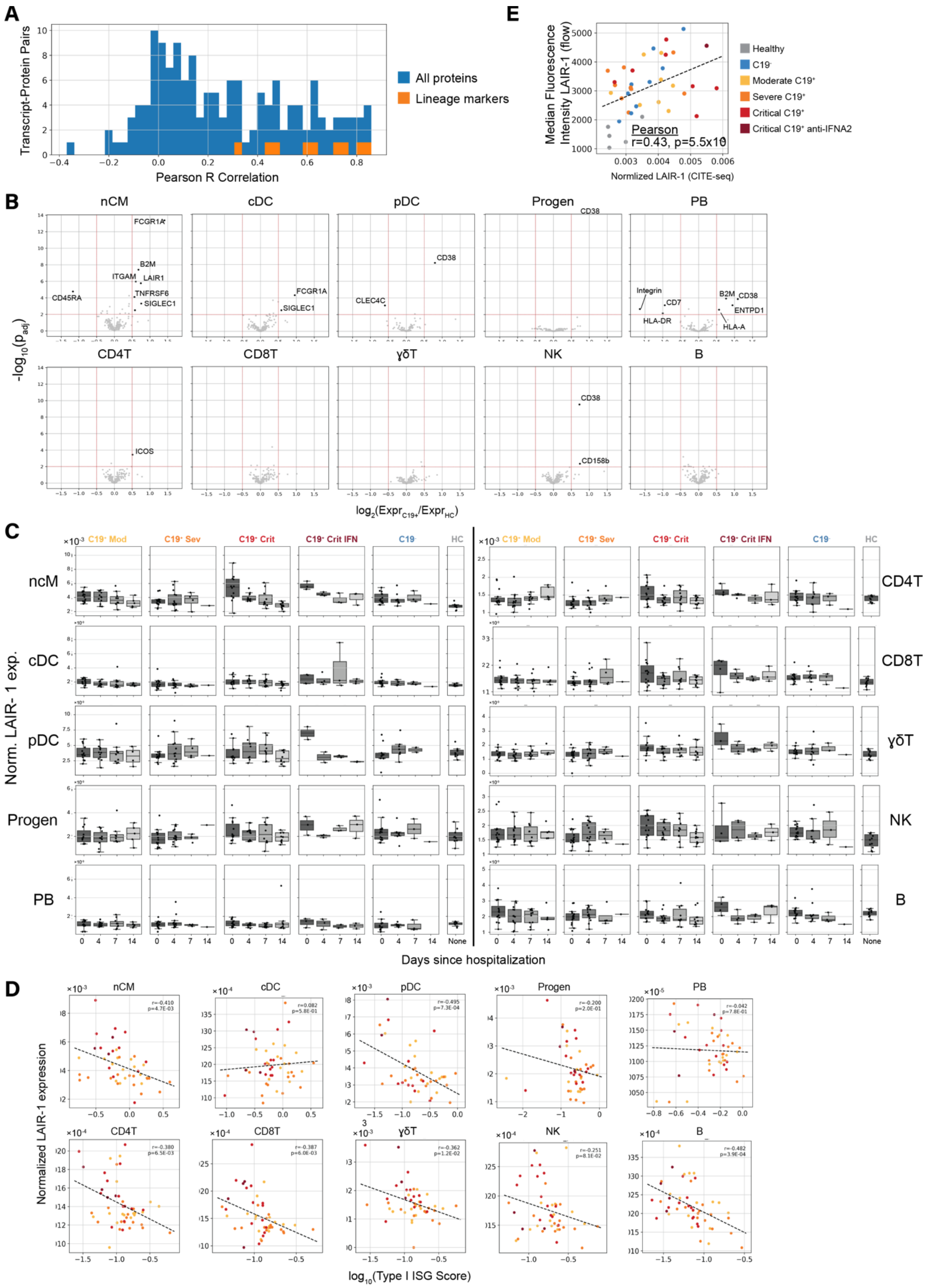
A
Transcript-Protein Pairs
Pearson R Correlation
All proteins
Lineage markers
E
Median Fluorescence Intensity LAIR-1 (flow)
Normlized LAIR-1 (CITE-seq)
Pearson
Healthy
C19-
Moderate C19+
Severe C19+
Critical C19+
Critical C19+ anti-IFNA2
B
nCM
cDC
pDC
Progen
PB
CD4T
CD8T
γδT
NK
B
FCGR1A
B2M
ITGAM
LAIR1
CD45RA
TNFRSF6
SIGLEC1
CLEC4C
CD38
Integrin
CD7
HLA-DR
ENTPD1
HLA-A
ICOS
CD158b
$-\log_{10}(p_{adj})$
$\log_2(Expr_{C19+}/Expr_{HC})$
C
C19+ Mod
C19+ Sev
C19+ Crit
C19+ Crit IFN
C19-
HC
Norm. LAIR- 1 exp.
Days since hospitalization
None
D
Normalized LAIR-1 expression
$\log_{10}$(Type I ISG Score)

Figure S6. LAIR1 surface protein abundance changes in leukocyte subsets in samples isolated from patients with critical COVID-19 and correlates with ISG response. (A) A density plot of Pearson R correlations is shown between normalized protein expression and corresponding transcript expression for each cell type in each sample. Correlations for 15 lineage markers are highlighted in orange. **(B)** Volcano plots of log fold change between COVID-19^{+} and healthy controls (x-axis) versus -log10(P-value) (y-axis) are shown for 10 additional cell types. Proteins that are statistically significant (FDR < 0.05) and have a log2(fold change) > 0.5 are highlighted. **(C)** Normalized leukocyte-associated immunoglobulin-like receptor 1 (LAIR1) surface expression (y-axis) for each cell type over the course of disease for healthy controls, COVID-19- controls, and COVID-19+ cases. COVID-19+ cases are separated by disease severity and the presence of anti-IFN-α2 antibodies. **(D)** Scatterplot of normalized (Norm.) LAIR1 expression (exp., y-axis) versus the type I-specific ISG score (x-axis) for additional cell types are shown, showing COVID-19+ cases colored by severity and anti-IFN-α2 status. **(E)** Scatterplots of LAIR1 protein expression of 40 samples collected at day 0 are shown using measurements from two different assays (CITE-seq and flow cytometry) showing a significant correlation of 0.43.
